# Supplementary material for: α-Melanocyte-stimulating hormone alleviates pathological cardiac remodeling via melanocortin 5 receptor
Source: EMBO Rep. 2024 Mar 7;25(4):21. doi: 10.1038/s44319-024-00109-6 (PMC11014855; doi:10.1038/s44319-024-00109-6)
Supplement: Supplementary file 1 — Appendix [file 44319_2024_109_MOESM1_ESM.pdf]

**α-Melanocyte-stimulating hormone alleviates pathological cardiac remodeling via melanocortin 5 receptor**

# Appendix - Table of Contents


| Appendix Item | Title | Page |
|---|---|---|
| Appendix Table S1 | Echocardiography in $Mc5r^{fl/fl}$, Myh6-MCM and Mc5r-cKO after 4 weeks of Ang II infusion or sham operation. | 2 |
| Appendix Table S2 | Ventricular weight and echocardiography in vehicle- and PG-901-treated C57Bl/6N mice after 5 weeks of sham operation. | 3 |
| Appendix Table S3 | Echocardiography in TAC-operated C57Bl/6J mice before the start of the drug intervention (vehicle or the α-MSH analogue) | 4 |
| Appendix Table S4 | Echocardiography in TAC-operated C57Bl/6N mice before the start of the drug intervention (vehicle or PG-901) | 4 |
| Appendix Table S5 | Quantitative RT-PCR primers for mouse genes | 5-6 |
| Appendix Table S6 | Quantitative RT-PCR primers for rat genes | 7 |
| Appendix Table S7 | Quantitative RT-PCR primers for human genes | 8 |
| Appendix Figure S1 | Chronic α-MSH treatment downregulates fibrosis-associated genes in the heart of TAC-operated mice. | 9 |
| Appendix Figure S2 | The effects of α-MSH on intracellular calcium signal and gene expression in cultured cardiomyocytes. | 10 |
| Appendix Figure S3 | The effects of the MC5-selective agonist PG-901 on intracellular calcium signal, cAMP level and gene expression in H9c2 cells. | 11 |
| Appendix Figure S4 | Inhibition of Gi/o or Gs signal transduction does not block the effects of PG-901. | 12 |
| Appendix Figure S5 | The effects of the MC5-selective agonist PG-901 on gene expression in H9c2 cells stimulated with Ang II, ET-1 or Phe. | 13 |
| Appendix Figure S6 | The effects of mechanical stretching and ET-1 on gene expression in hiPSC-CMs. | 14 |
| Appendix Figure S7 | Generation of tamoxifen-inducible cardiomyocyte-specific MC5-R knockout (Mc5r-cKO) mice. | 15 |
| Appendix Figure S8 | Echocardiography and cardiac gene expression profiling in cardiomyocyte-specific MC5-R knockout (Mc5r-cKO) mice. | 16 |
| Appendix Figure S9 | Cardiomyocyte-specific MC5-R deficiency enhances apoptosis in the heart of TAC-operated mice. | 17 |
| Appendix Figure S10 | Cardiomyocyte-specific MC5-R deficiency does not affect Ang II-induced cardiac hypertrophy. | 18 |
| Appendix Figure S11 | The effects of PG-901 on LV end-diastolic dimension, diastolic function and cardiac gene expression in TAC-operated mice. | 19 |

**Appendix Table S1**: Echocardiography in $Mc5r^{fl/fl}$, Myh6-MCM and Mc5r-cKO after 4 weeks of Ang II infusion or sham operation.

| Parameter | | $Mc5r^{fl/fl}$ Ang II (n=9) Sham (n=5) | Myh6-MCM Ang II (n=6) Sham (n=3) | Mc5r-cKO Ang II (n=8) Sham (n=4) |
|---|---|---|---|---|
| **LVEDD (mm)** | Ang II | 3.87 ± 0.11 | 4.02 ± 0.22 | 3.92 ± 0.09 |
| | Sham | 4.11 ± 0.06 | 4.11 ± 0.09 | 3.98 ± 0.06 |
| **LVPW (mm)** | Ang II | 0.92 ± 0.06 | 0.86 ± 0.07 | 0.97 ± 0.05 |
| | Sham | 0.67 ± 0.02 | 0.59 ± 0.01 | 0.69 ± 0.02 |
| **EF (%)** | Ang II | 58.5 ± 2.5 | 59.7 ± 6.0 | 55.7 ± 5.8 |
| | Sham | 50.9 ± 2.7 | 56.1 ± 1.8 | 55.5 ± 2.7 |
| **FS (%)** | Ang II | 30.6 ± 1.7 | 32.5 ± 4.4 | 29.9 ± 4.1 |
| | Sham | 25.7 ± 1.6 | 29.0 ± 1.2 | 28.6 ± 1.8 |
| **FAC (%)** | Ang II | 42.7 ± 2.7 | 44.1 ± 4.3 | 40.3 ± 4.6 |
| | Sham | 41.0 ± 3.2 | 34.0 ± 1.1 | 40.9 ± 1.8 |

Data are mean ± SEM. LVEDD indicates LV end-diastolic dimension; LVPW, LV posterior wall thickness (diastole); EF, ejection fraction; FS, fractional shortening; FAC, fractional area change.

**Appendix Table S2**: Ventricular weight and echocardiography in vehicle- and PG-901-treated C57Bl/6N mice after 5 weeks of sham operation.

| **Parameter** | **Sham/Vehicle** n=8 | **Sham/PG-901** 0.005 mg/kg n=8 | **Sham/PG-901** 0.5 mg/kg n=7 |
|---|---|---|---|
| **VW (mg)** | 130.8 ± 2.7 | 129.7 ± 3.7 | 122.9 ± 2.0 |
| **VW/BW (mg/g)** | 4.7 ± 0.1 | 4.6 ± 0.1 | 4.6 ± 0.1 |
| **VW/TL (mg/mm)** | 7.6 ± 0.2 | 7.5 ± 0.2 | 7.1 ± 0.1 |
| **LVEDD (mm)** | 4.33 ± 0.06 | 4.48 ± 0.12 | 4.26 ± 0.11 |
| **LVPW (mm)** | 0.69 ± 0.03 | 0.66 ± 0.02 | 0.64 ± 0.03 |
| **EF (%)** | 50.8 ± 2.6 | 47.0 ± 4.5 | 53.5 ± 2.3 |
| **FS (%)** | 25.8 ± 1.6 | 24.0 ± 2.9 | 27.5 ± 1.5 |

Data are mean ± SEM. VW indicates ventricular weight; VW/BW indicates ventricular weight-to-body weight ratio; VW/TL indicates ventricular weight-to-tibia length ratio; LVEDD, LV end-diastolic dimension; LVPW, LV posterior wall thickness (diastole); EF, ejection fraction; FS, fractional shortening.

**Appendix Table S3**: Echocardiography in TAC-operated C57Bl/6J mice before the start of the drug intervention (vehicle or the α-MSH analogue)

| Parameter | TAC/Vehicle n=13 | TAC/α-MSH n=10 | P-value |
|---|---|---|---|
| **LVEDD (mm)** | 4.61 ± 0.10 | 4.49 ± 0.10 | 0.42 |
| **LVPW (mm)** | 0.53 ± 0.02 | 0.54 ± 0.02 | 0.71 |
| **EF (%)** | 50.1 ± 4.6 | 47.5 ± 1.6 | 0.58 |
| **FS (%)** | 25.5 ± 2.4 | 25.1 ± 2.8 | 0.92 |

Data are mean ± SEM. LVEDD indicates LV end-diastolic dimension; LVPW, LV posterior wall thickness (diastole); EF, ejection fraction; FS, fractional shortening.

**Appendix Table S4**: Echocardiography in TAC-operated C57Bl/6N mice before the start of the drug intervention (vehicle or PG-901)

| Parameter | TAC/Vehicle n=11 | TAC/PG-901 0.005 mg/kg n=9 | TAC/PG-901 0.5 mg/kg n=7 | P-value Vs. Vehicle 0.005/0.5 mg/kg |
|---|---|---|---|---|
| **LVEDD (mm)** | 4.62 ± 0.06 | 4.55 ± 0.06 | 4.60 ± 0.08 | 0.46 / 0.87 |
| **LVPW (mm)** | 0.53 ± 0.01 | 0.50 ± 0.02 | 0.50 ± 0.02 | 0.22 / 0.25 |
| **EF (%)** | 46.0 ± 1.6 | 45.7 ± 2.0 | 45.0 ± 1.7 | 0.90 / 0.68 |
| **FS (%)** | 23.0 ± 0.9 | 22.7 ± 1.1 | 22.3 ± 1.0 | 0.88 / 0.67 |

Data are mean ± SEM. LVEDD indicates LV end-diastolic dimension; LVPW, LV posterior wall thickness (diastole); EF, ejection fraction; FS, fractional shortening.

**Appendix Table S5**: Quantitative RT-PCR primers for mouse genes

| Gene name (accession number) | 5'-3' primer sequence |
|---|---|
| *Acta1* (NM_001272041.1) | Fwd: cccaaagctaaccgggagaag |
| | Rev: ccagaatccaacacgatgcc |
| *Acta2* (NM_007392.3) | Fwd: agattgtgcgcgacatcaaag |
| | Rev: gcagactccataccgataaagga |
| *Actb* (NM_007393.5) | Fwd: tccatcatgaagtgtgacgt |
| | Rev: gagcaatgatcttgatcttca |
| *Bax* (NM_007527.4) | Fwd: aaactggtgctcaaggcc |
| | Rev: cttggatccagacaagcagc |
| *Casp3* (NM_009810.3) | Fwd: tggtgatgaaggggtcatttatg |
| | Rev: ttcggctttccagtcagactc |
| *Col1a1* (NM_007742.4) | Fwd: gctcctcttaggggccact |
| | Rev: ccacgtctcaccattgggg |
| *Col1a2* (NM_007743.3) | Fwd: tgcagtaacttcgtgcctagc |
| | Rev: acgtggtcctctgtctcca |
| *Col3a1* (NM_009930.2) | Fwd: ctaaaattctgccaccccgaa |
| | Rev: aggatcaacccagtattctccactc |
| *Ctgf* (NM_010217.2) | Fwd: agacctgtgggatgggcat |
| | Rev: gcttggcgattttaggtgtcc |
| *Fn1* (NM_010233.2) | Fwd: atgtggacccctcctgatagt |
| | Rev: gcccagtgatttcagcaaagg |
| *Il6* (NM_031168.2) | Fwd: ggccttccctacttcacaag |
| | Rev: atttccacgatttcccagag |
| *Mc5r* (NM_013596.2) | Fwd: caagaccagagcccggtaaac |
| | Rev: gcgcaaaggtaagcatgattct |
| *Mmp2* (NM_008610.3) | Fwd: gatgtcgcccctaaaacagac |
| | Rev: cagccatagaaagtgttcaggt |
| *Noxa* (NM_021451.2) | Fwd: gcagagctaccacctgagttc |
| | Rev: cttttgcgacttcccaggca |
| *Nppa (NM_008725.3)* | Fwd: gcttccaggccatattggag |
| | Rev: gggggcatgacctcatctt |
| *Nppb* (NM_008726.6) | Fwd: cccaaaaagagtccttcggtc |
| | Rev: cggtctatcttgtgcccaaag |

**Appendix Table S5**: Quantitative RT-PCR primers for mouse genes

| **Gene name (accession number)** | **5'-3' primer sequence** |
|---|---|
| *Pomc* (NM_008895.4) | Fwd: caagccggtgggcaagaaacg |
| | Rev: ctaatggccgctcgccttccag |
| *Serca2a* (NM_001110140.3) | Fwd: gagaacgctcacacaaagacc |
| | Rev: cttcttcagccggcaattcgttg |
| *Tgfb1* (NM_011577.2) | Fwd: ccgcaacaacgccatctatg |
| | Rev: cccgaatgtctgacgtattgaag |

**Appendix Table S6**: Quantitative RT-PCR primers for rat genes

| Gene name (accession number) | 5’-3’ primer sequence |
|---|---|
| *Acta1* (NM_019212.3) | Fwd: tgaagcctcacttcctaccc |
| | Rev: cgtcacacatggtgtctagttc |
| *Acta2* (NM_031004.2) | Fwd: actgggacgacatggaaaag |
| | Rev: catctccagagtccagcaca |
| *Col1a1* (NM_053304.1) | Fwd: gcgaaggcaacagtcgattc |
| | Rev: cccaagttccggtgtgactc |
| *Col1a2* (NM_053356.2) | Fwd: ctggatttgctggcgagaag |
| | Rev: aataccgggagcaccaagaag |
| *Col3a1* (NM_032085.1) | Fwd: cagctggccttcctcagactt |
| | Rev: gctgtttttgcagtggtatgtaatg |
| *Ctgf* (NM_022266.2) | Fwd: gaggaaaacattaagaagggcaaa |
| | Rev: cggcacaggtcttgatga |
| *Fn1* (NM_019143.2) | Fwd: gctgctgggacttccacgt |
| | Rev: tctgttccgggaggtgca |
| *Gapdh* (NM_017008.4) | Fwd: gacatgccgcctggagaaac |
| | Rev: agcccaggatgccctttagt |
| *Il6* (NM_012589.2) | Fwd: cctggagtttgtgaagaacaact |
| | Rev: ggaagttggggtaggaagga |
| *Mc5r* (NM_013182.3) | Fwd: ccagcatgaagggtgctatc |
| | Rev: gggaccagcagacaatgaa |
| *Mmp2* (NM_031054.2) | Fwd: aaaggagggctgcattgtgaa |
| | Rev: ctggggaaggacgtgaagagg |
| *Nppb* (NM_031545.1) | Fwd: acaatccacgatgcagaagct |
| | Rev: gggccttggtcctttgaga |
| *Rn18s* (NR_046237.2) | Fwd: cattcgaacgtctgccctat |
| | Rev: gtttctcaggctccctctcc |
| *Tgfb1* (NM_021578.2) | Fwd: gcaacaacgcaatctatgac |
| | Rev: cctgtattccgtctcctt |

**Appendix Table S7**: Quantitative RT-PCR primers for human genes

| Gene name (accession number) | 5'-3' primer sequence |
|---|---|
| *ACTB* (NM_001101.5) | Fwd: caccattggcaatgagcggttc |
| | Rev: aggtctttgcggatgtccacgt |
| *ACTA1* (NM_001100.4) | Fwd: aggtcatcaccatcggcaacga |
| | Rev: gctgttgtaggtggtctcgtga |
| *ACTA2* (NM_001613.4) | Fwd: ctatgcctctggacgcacaact |
| | Rev: cagatccagacgcatgatggca |
| *CTGF* (NM_001901.4) | Fwd: tcccaaaatctccaagccta |
| | Rev: gtaatggcaggcacaggtct |
| *FN1* (NM_212482.4) | Fwd: cggtggctgtcagtcaaag |
| | Rev: aaacctcggcttcctccataa |
| *GAPDH* (NM_002046.7) | Fwd: tcaaggctgagaacgggaag |
| | Rev: cgccccacttgattttggag |
| *MC5R* (NM_005913.3) | Fwd: ttggatctcaacctgaatgcc |
| | Rev: gcccctatgaccaagatgttctc |
| *NPPA* (NM_006172.4) | Fwd: acaatgccgtgtccaacgcaga |
| | Rev: cttcattcggctcactgagcac |
| *NPPB* (NM_002521.3) | Fwd: tctggctgctttgggaggaaga |
| | Rev: ccttgtggaatcagaagcaggtg |
| *RPS18* (NM_022551.3) | Fwd: cgccgctagaggtgaaattc |
| | Rev: ccagtcggcatcgtttatgg |
| *POMC* (NM_000939.4) | Fwd: cgcccagtgaaggtgtaccc |
| | Rev: ggcgtctggctcttctcggaggtc |
| *TGFB1* (NM_000660.7) | Fwd: tacctgaacccgtgttgctctc |
| | Rev: gttgctgaggtatcgccaggaa |

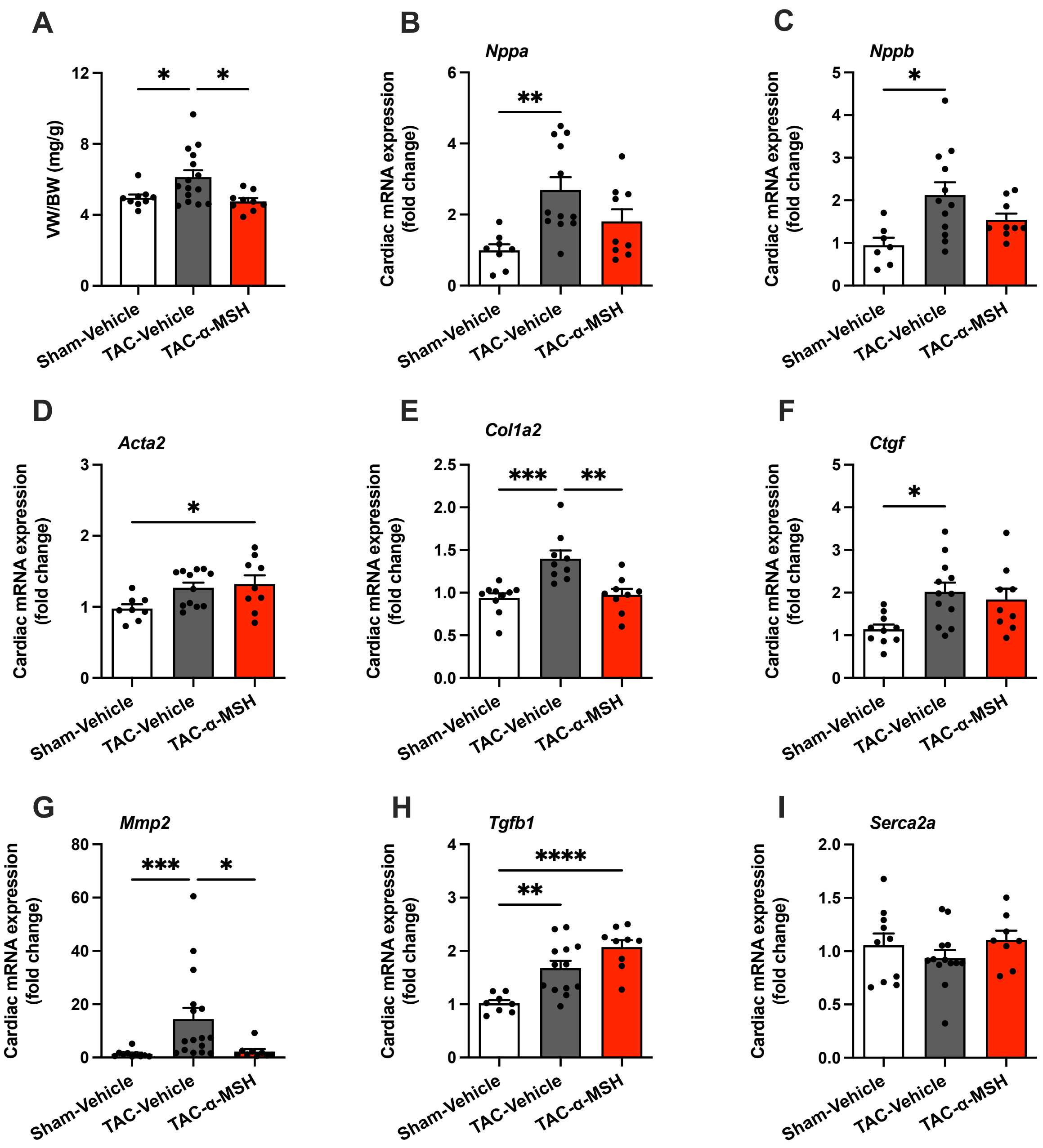


**Appendix Figure S1. Chronic α-MSH treatment downregulates fibrosis-associated genes in the heart of TAC-operated mice.** (**A**) Ventricular weight to body weight ratio (VW/BW) in sham- and TAC-operated mice treated with either vehicle or α-MSH analogue. (**B-I**) The expression of *Nppa*, *Nppb*, *Acta2*, *Col1a2*, *Ctgf*, *Mmp2*, *Tgfb1* and *Serca2a* mRNA in the LV of sham- and TAC-operated mice. Data are mean ± SEM, *n*=8-12 mice per group. * $P<0.05$, ** $P<0.01$, *** $P<0.001$ and **** $P<0.0001$ for the indicated comparisons by 1-way ANOVA and Dunnett *post hoc* tests.

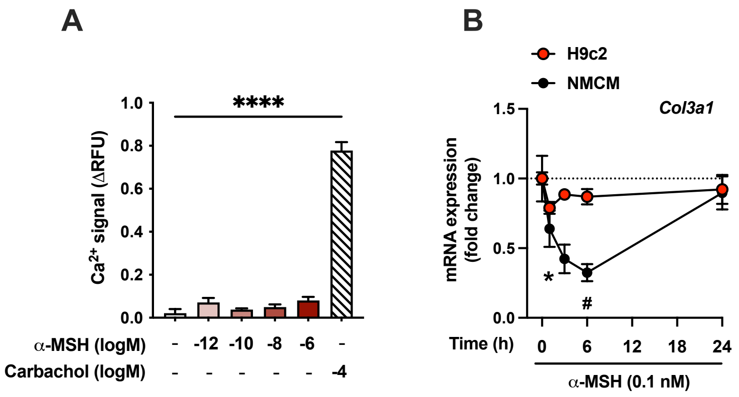


**Appendix Figure S2**. **The effects of α-MSH on intracellular calcium signal and gene expression in cultured cardiomyocytes**. (**A**) Intracellular calcium signal in response to α-MSH and carbachol (positive control) in H9c2 cells. **** *P*<0.0001 by 1-way ANOVA and Dunnett *post hoc* test. (**B, C**) Gene expression analysis of *Col3a1* and *Fn1* in H9c2 cells and NMCMs treated with α-MSH (0.1 nM) for 1-24 hours. Data are mean ± SEM, *n*=4-6 per group. * *P*<0.05 versus Control (0 h) in H9c2 cells, # *P*<0.05 versus Control (0 h) in NMCMs by 1-way ANOVA and Dunnett *post hoc* tests.

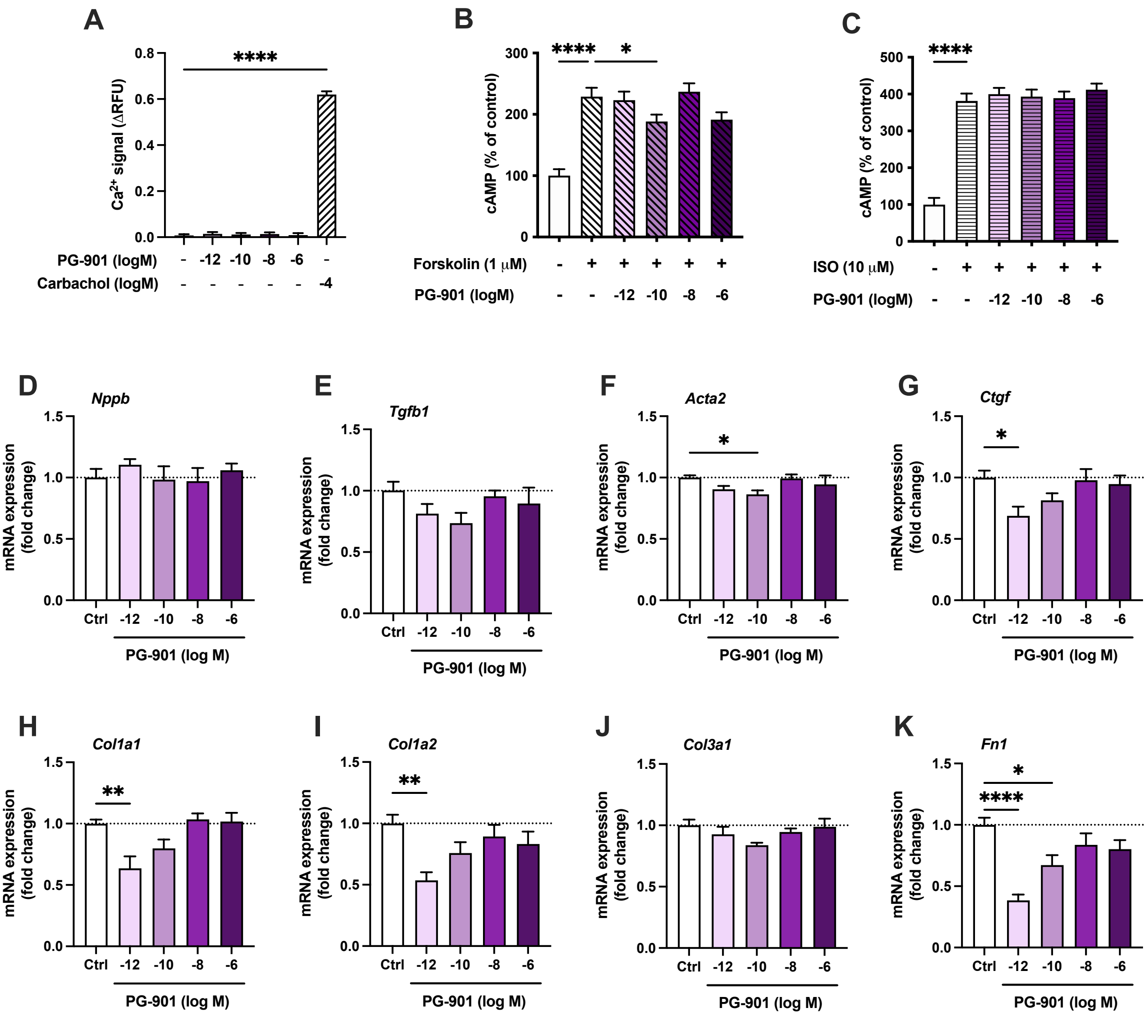


**Appendix Figure S3**. **The effects of the MC5-selective agonist PG-901 on intracellular calcium signal, cAMP level and gene expression in H9c2 cells**. (**A**) Intracellular calcium signal in response to PG-901 and carbachol (positive control). **** *P*<0.0001 versus Control by 1-way ANOVA and Dunnett *post hoc* tests (**B, C)** The effects of PG-901 on intracellular cAMP levels in forskolin- and isoprenaline -stimulated H9c2 cells. *n*=6-10 per group. * *P*<0.05 and **** *P*<0.0001 for the indicated comparisons by 1-way ANOVA and Dunnett *post hoc* tests. (**D**-**K**) Quantitative real-time PCR (qPCR) analysis of *Nppb* (brain natriuretic peptide), *Tgfb1* (transforming growth factor beta 1), *Acta2* (alpha-smooth muscle actin), *Ctgf* (connective tissue growth factor), *Col1a1* (collagen type I, alpha 1), *Col1a2* (collagen type I, alpha 2), *Col3a1* (collagen type III, alpha 1) and *Fn1* (fibronectin) in H9c2 cells treated with different concentrations of PG-901 for 3 hours. * *P*<0.05, ** *P*<0.01 and **** *P*<0.0001 versus Control by 1-way ANOVA and Dunnett *post hoc* tests, *n*=4-6 per group in each graph. Data are mean ± SEM.

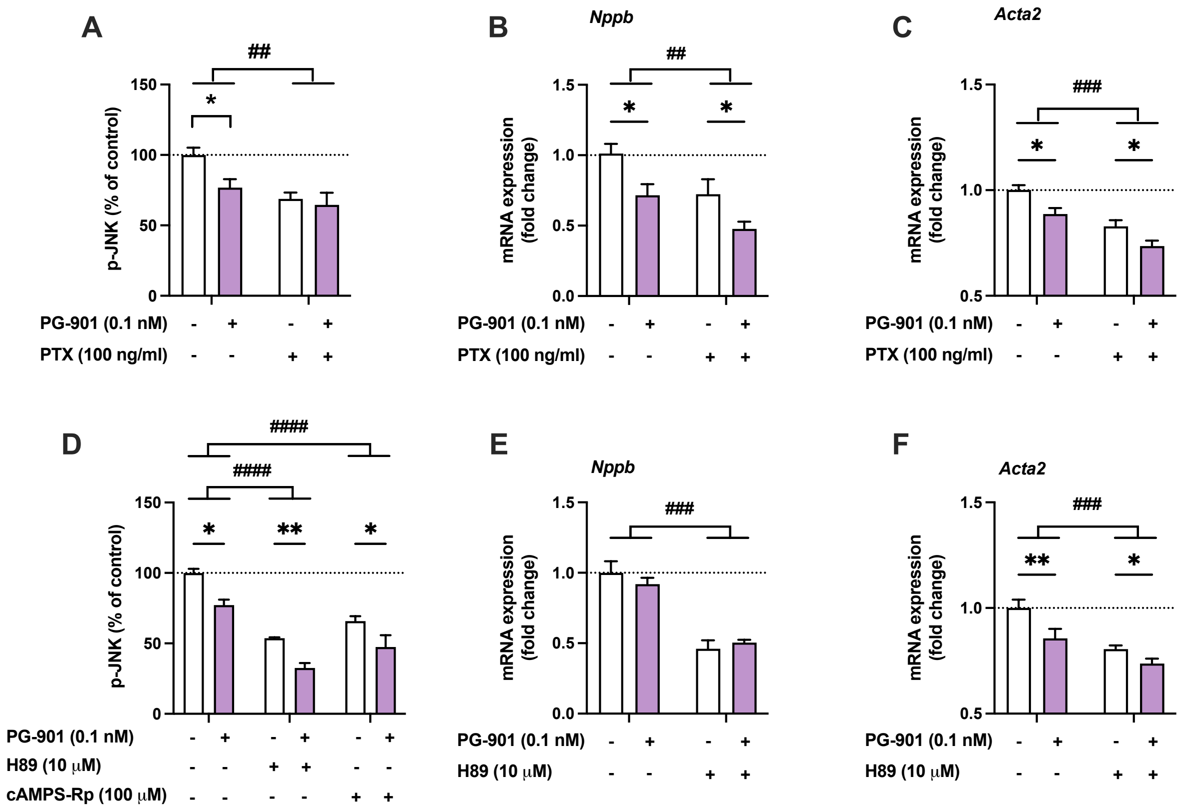


**Appendix Figure S4. Inhibition of Gi/o or Gs signal transduction does not block the effects of PG-901.** (**A**) Quantification of p-JNK (normalized to total protein expression) level in H9c2 cells treated with or without pertussis toxin (PTX) for 18 hours followed by PG-901 treatment for 60 minutes. p-JNK expression was analyzed by an ELISA assay. (**B, C**) Quantitative real-time PCR (qPCR) analysis of *Nppb* and *Acta2* mRNA expression in H9c2 cells treated with or without PTX for 18 hours followed by PG-901 treatment for 3 hours. (**D**) Quantification of p-JNK (normalized to total protein expression) level by ELISA assay in H9c2 treated with the PKA inhibitor H89 (10 μM) or cAMPS-Rp (100 μM) for 30 minutes followed by PG-901 treatment for 60 minutes. (**E, F**) Quantitative real-time PCR (qPCR) analysis of *Nppb* and *Acta2* mRNA expression in H9c2 cells treated with or without H89 (10 μM) for 30 minutes followed by PG-901 treatment for 3 hours. Data are mean ± SEM, *n*=5-6 per group in each graph. * $P$<0.05, ** $P$<0.01 for the indicated *post hoc* comparisons. ## $P$<0.01, ### $P$<0.001, #### $P$<0.0001 for the main effect of PTX/H-89/ cAMPS-Rp by 2-way ANOVA.

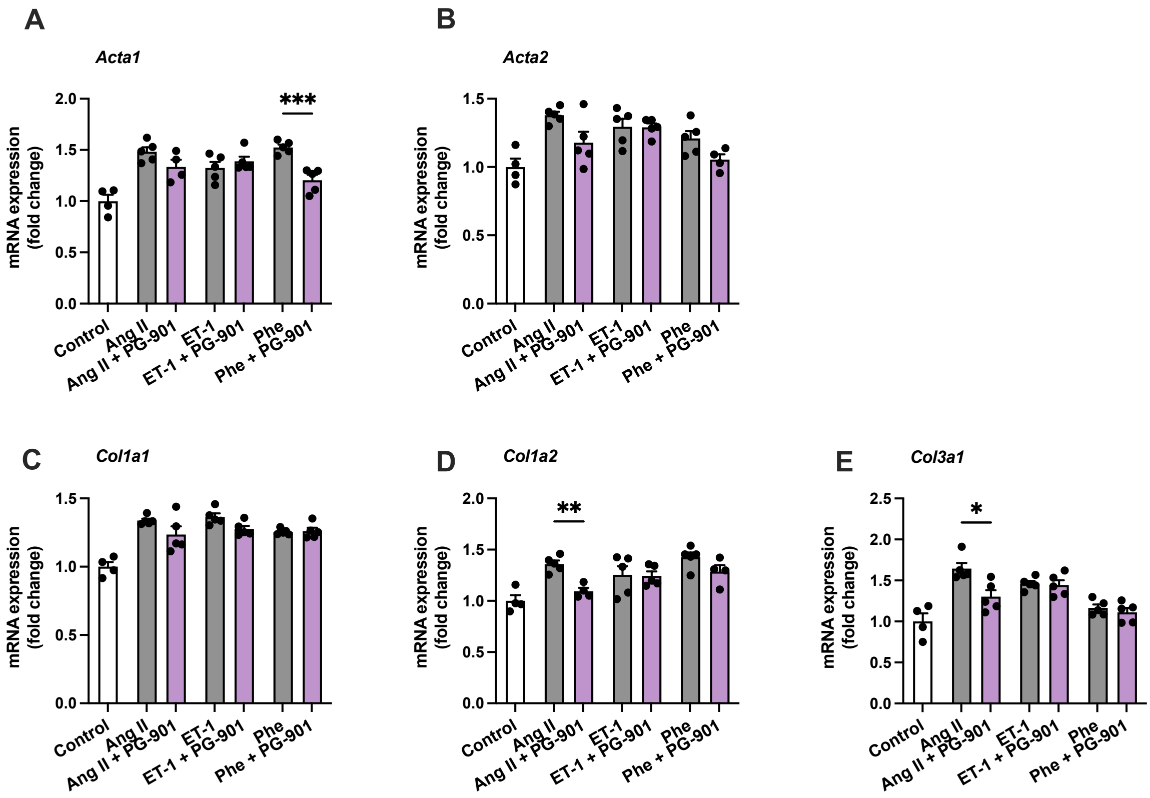


**Appendix Figure S5. The effects of the MC5-selective agonist PG-901 on gene expression in H9c2 cells stimulated with Ang II, ET-1 or phenylephrine**. (**A-E**) Quantitative real-time PCR (qPCR) analysis of *Acta1*, *Acta2, Col1a1*, *Col1a2 and Col3a1* in H9c2 cells treated with angiotensin II (Ang II, 0.1 μM), endothelin-1 (ET-1, 0.1 μM) or phenylephrine (Phe, 0.1 mM) for 3 hours in the absence or presence of PG-901 (0.1 nM). * *P*<0.05, ** *P*<0.01 and *** *P*<0.001 for the indicated comparisons by 1-way ANOVA and Dunnett *post hoc* tests, *n*=4-5 per group in each graph. Data are mean ± SEM.

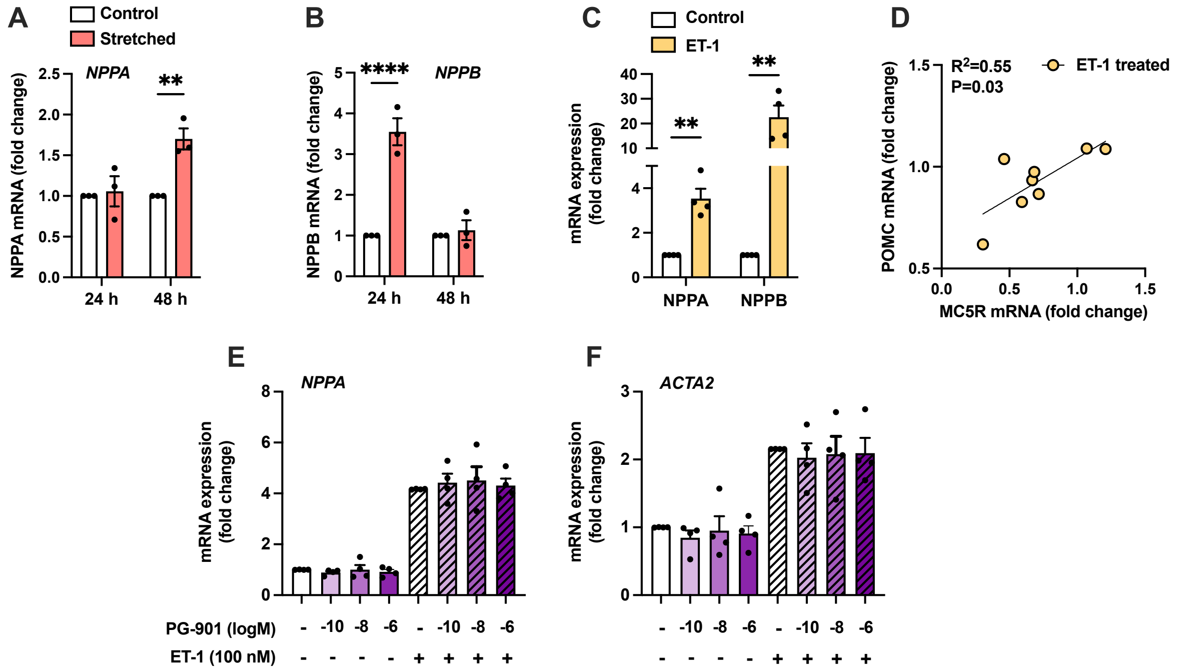


**Appendix Figure S6. The effects of mechanical stretching and endothelin 1 on gene expression in human induced pluripotent stem cell-derived cardiomyocytes (hiPSC-CM).** (**A**, **B**) Quantitative real-time PCR (qPCR) analysis of atrial natriuretic peptide (*NPPA*) and B-type natriuretic peptide (*NPPB*) mRNA expression in human induced pluripotent stem cell-derived cardiomyocytes (hiPSC-CMs) that were mechanically stretched for 24 or 48 hours. *n*=3 individual experiments/batches of differentiation. ** *P*<0.01 and **** *P*<0.0001 versus Control by randomized block ANOVA (using individual experiments and treatment as factors). (**C**) qPCR analysis of *NPPA* and *NPPB* mRNA expression in hiPSC-CMs treated with endothelin 1 (ET-1, 100 nM) for 24 hours. *n*=4 individual experiments/batches of differentiation. (**D**) Correlation between MC5R and POMC mRNA expression in ET-1 treated hiPSC-CM samples. Coefficient of determination (R squared; R2) and *P* value by Pearson correlation are presented in the graph. *n*=8 from 4 individual experiments/batches of differentiation. (**E**, **F**) qPCR analysis of *NPPA* and *ACTA2* mRNA expression in hiPSC-CMs treated with different concentrations of PG-901 for 24 hours in the absence or presence of ET-1 (100 nM). *n*=4 individual experiments/batches of differentiation.

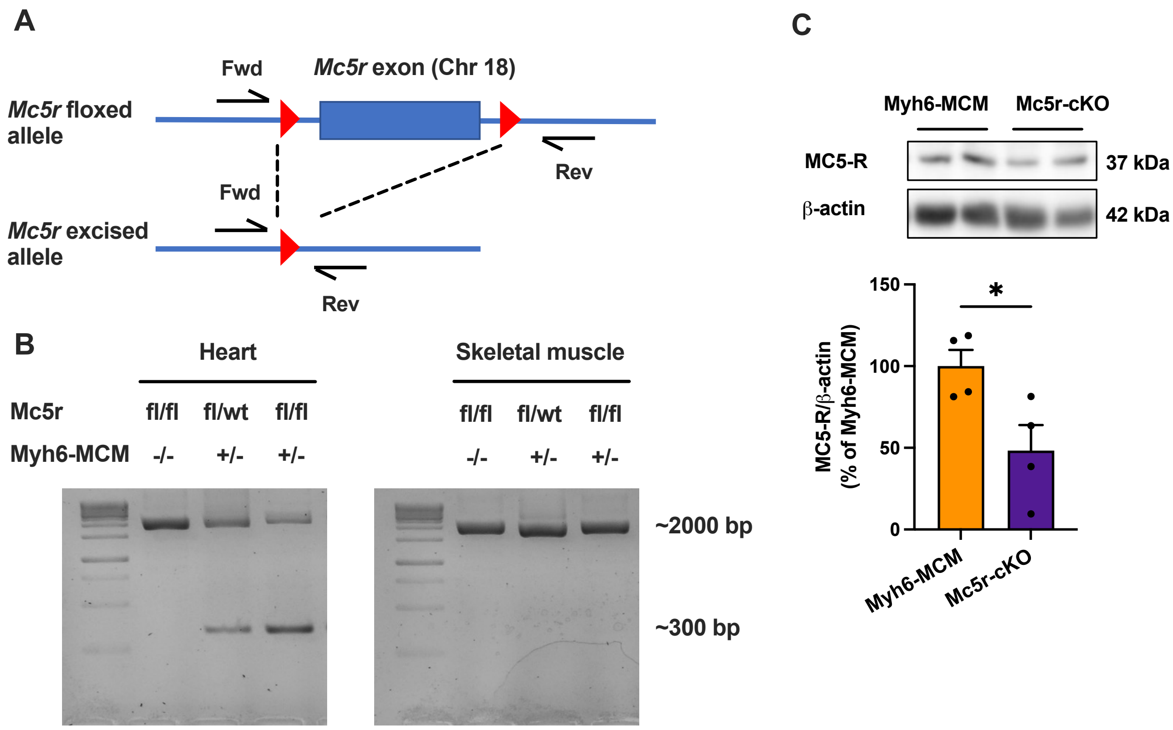


**Appendix Figure S7. Generation of tamoxifen-inducible cardiomyocyte-specific MC5-R knockout (Mc5r-cKO) mice.** (**A**) Schematic presentation of the loxP-flanked (floxed) Mc5r allele and the excised *Mc5r* allele after Myh6-MCM -mediated recombination. Fwd and Rev indicate forward and reverse primer positions used for PCR genotyping. Red arrow heads indicate loxP-sites. (**B**) PCR analysis of genomic DNA extracted from the left ventricle (LV) and skeletal muscle (quadriceps femoris) of Cre-negative Mc5r$^{fl/fl}$ mouse, Cre-positive heterozygous Mc5r$^{fl/wt}$ mouse and Cre-positive homozygous Mc5r$^{fl/fl}$ mouse at 12 weeks of age and 6 weeks after tamoxifen treatment. The size of the recombined allele is ~300 bp. (**C**) Representative Western blot and quantification of relative MC5-R protein expression (normalized to β-actin) in the LV of Cre-positive wildtype Mc5r$^{wt/wt}$ (Myh6-MCM) and Cre-positive homozygous Mc5r$^{fl/fl}$ mice (Mc5r-cKO). *n*=4 mice per group, each dot represents individual mouse. Data are mean ± SEM, * *P*<0.05 versus control mice by Student's t test.

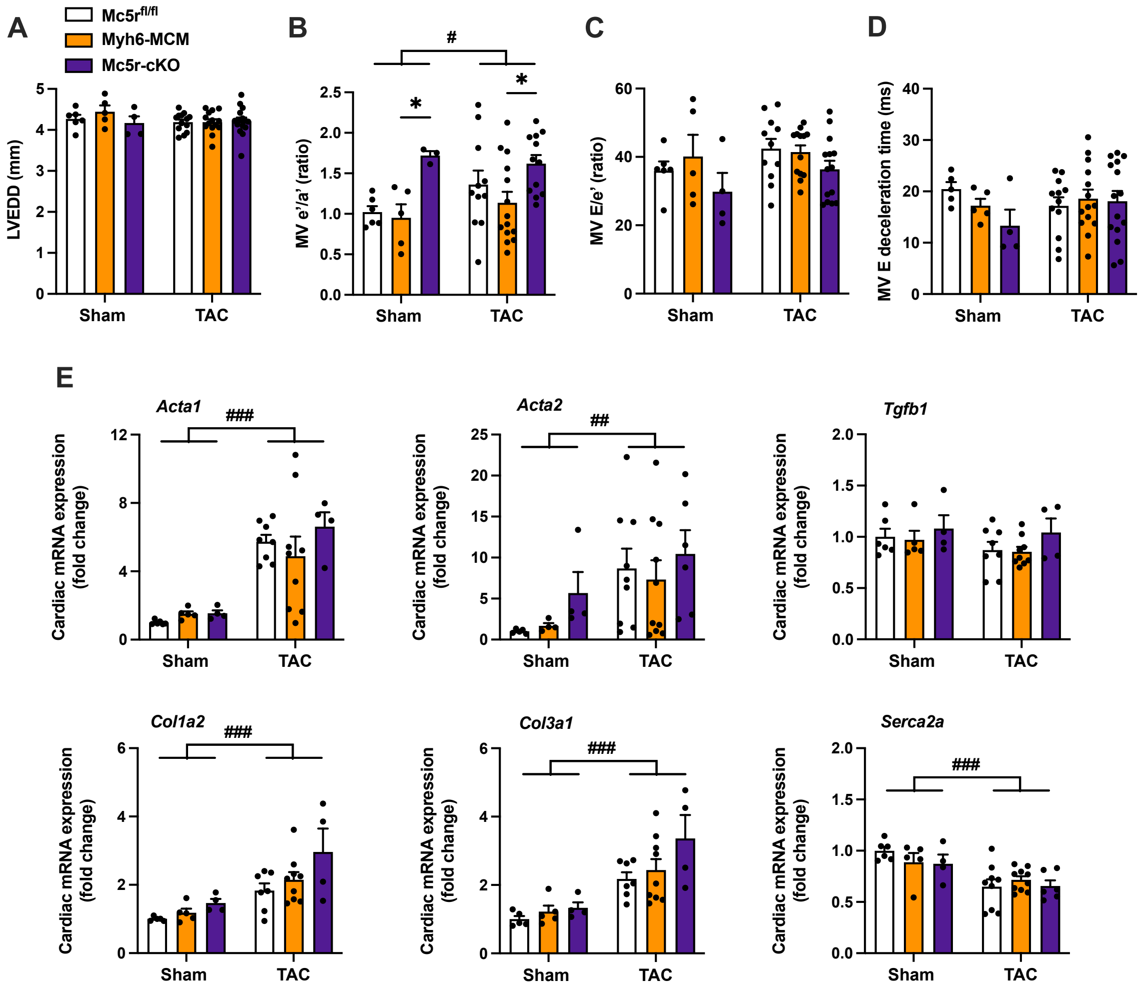


**Appendix Figure S8. Echocardiography and cardiac gene expression profiling in cardiomyocyte-specific MC5-R knockout (Mc5r-cKO) mice.** (**A-D**) Echocardiographic analysis of LV end-diastolic dimension (LVEDD) and mitral annular e'/a' ratio, E/e' ratio and mitral valve E wave (MV E) deceleration time in Mc5r$^{fl/fl}$, Myh6-MCM and Mc5r-cKO mice after 4 weeks of sham or TAC. (**E**) Quantitative real-time PCR (qPCR) analysis of fibrotic marker genes (*Acta1*, *Acta2*, *Tgfb1*, *Col1a2* and *Col3a1*,) and *Serca2a* after sham or TAC surgery in the LV of Mc5r$^{fl/fl}$, Myh6-MCM and Mc5r-cKO mice. Data are mean ± SEM, *n*=4-10 mice per group. * *P*<0.05 for the indicated comparisons by 2-way ANOVA and Dunnett's *post hoc* tests. # *P*<0.05, ## *P*<0.01, ### *P*<0.001 for the main effect of TAC by 2-way ANOVA.

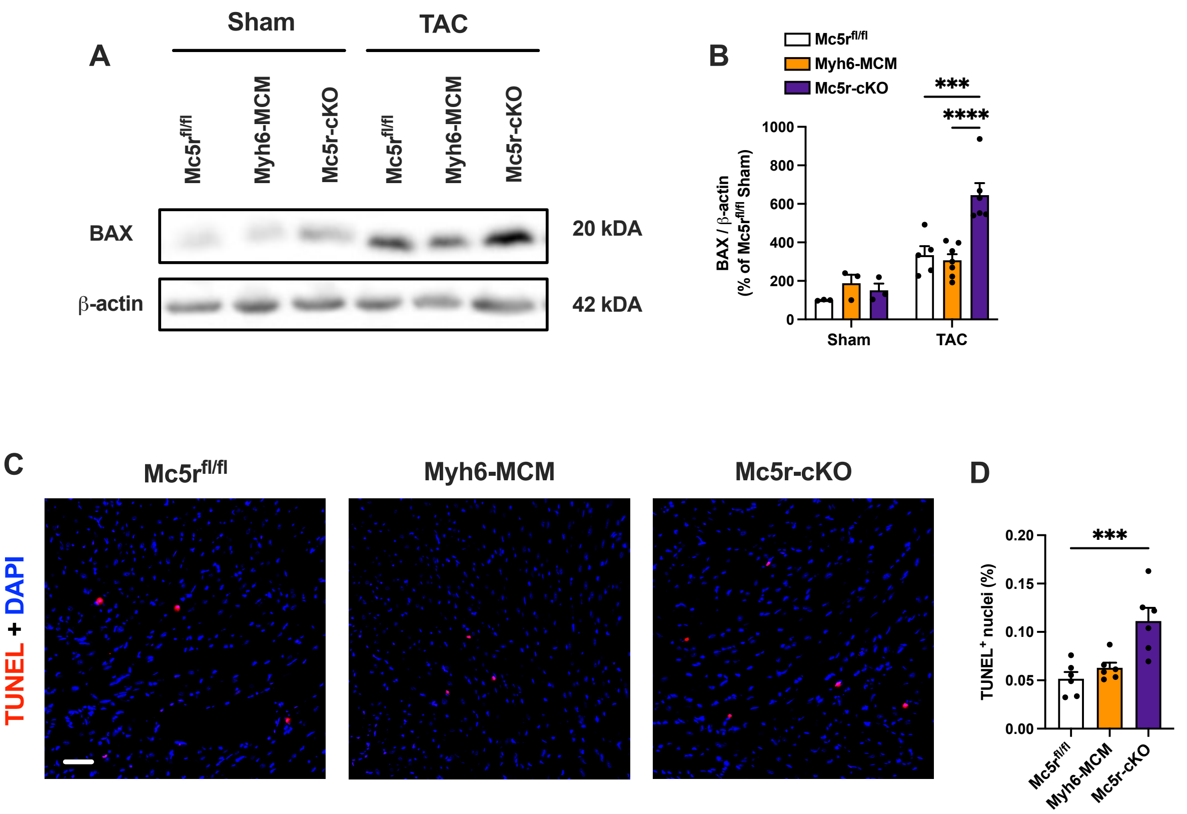


**Appendix Figure S9**. **Cardiomyocyte-specific MC5-R deficiency enhances apoptosis in the heart of TAC-operated mice.** (**A, B**) Representative Western blot and quantification of relative BAX protein expression (normalized to β-actin) in the LV of Mc5r$^{fl/fl}$, Myh6-MCM and Mc5r-cKO mice after 4 weeks of sham or TAC operation. *n*=3-7 mice per group. *** *P*<0.001 and **** *P*<0.0001 for the indicated comparisons by 2-way ANOVA and Dunnett's *post hoc* tests (**C, D**) Representative images and quantitative analysis showing relative amount of apoptotic TUNEL-positive nuclei in the LV of TAC-operated Mc5r$^{fl/fl}$, Myh6-MCM and Mc5r-cKO mice. Scale bar, 50 μm. *n*=6 mice per group. *** *P*<0.001 for the indicated comparisons by 1-way ANOVA and Dunnett's *post hoc* tests. Data are mean ± SEM.

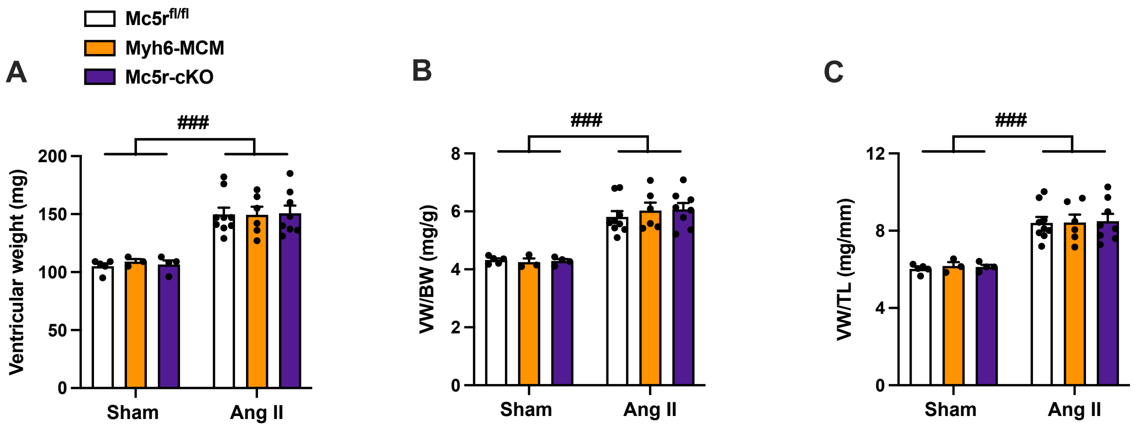


**Appendix Figure S10**. **Cardiomyocyte-specific MC5-R deficiency does not affect Ang II-induced cardiac hypertrophy**. (**A**-**C**) Ventricular weight, ventricular weight to body weight ratio (VW/BW) and ventricular weight to tibia length ratio (VW/TL) in Mc5r$^{fl/fl}$, Myh6-MCM and Mc5r-cKO mice after sham operation or Ang II infusion for 4 weeks. Data are mean ± SEM, *n*=3-9 mice per group. Each dot represents individual mouse. ### *P*<0.001 for the main effect of Ang II by 2-way ANOVA.

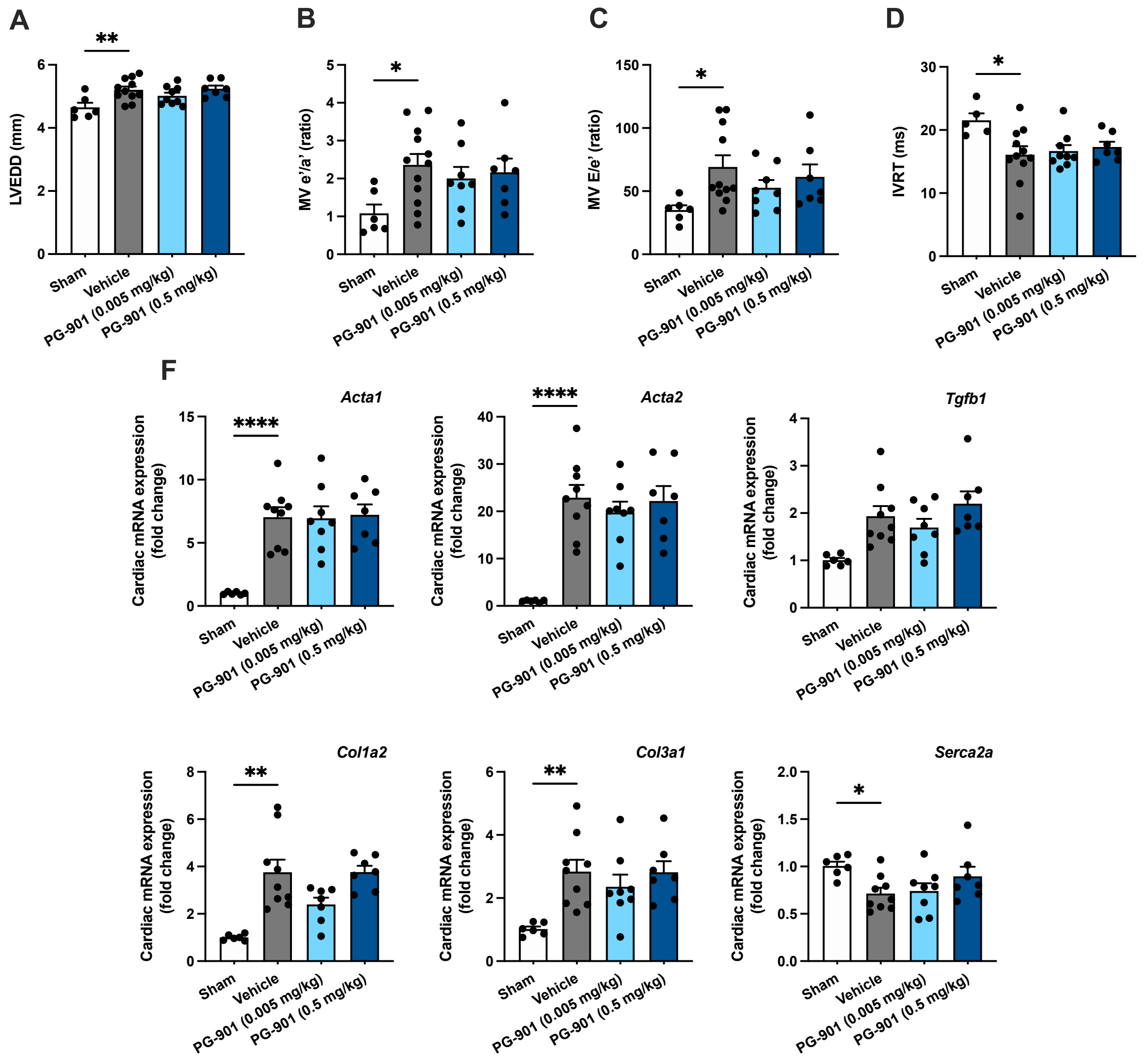


**Appendix Figure S11. The effects of PG-901 on LV end-diastolic dimension, diastolic function and cardiac gene expression in TAC-operated mice**. (**A-D**) Left ventricular end-diastolic dimension (LVEDD), mitral annular e'/a' ratio, E/e' ratio and isovolumetric relaxation time (IVRT) in sham- and TAC-operated mice treated with either vehicle or PG-901 (0.5 or 0.005 mg/kg/day) at the end of the experiment. (**E**) Left ventricular end-diastolic dimension (LVEDD) analyzed by echocardiography at the end of the experiment (**F**) Quantitative real-time PCR (qPCR) analysis of *Acta1*, *Acta2*, *Tgfb1*, *Col1a2*, *Col3a1* and *Serca2a* in the LV of sham- and TAC-operated mice treated with either vehicle or PG-901. Data are mean ± SEM, each dot represents individual mouse. * *P*<0.05, ** *P*<0.01 and **** *P*<0.0001 for the indicated comparisons by 1-way ANOVA and Dunnett *post hoc* tests.
