# Supplementary figures and images for: α-Melanocyte-stimulating hormone alleviates pathological cardiac remodeling via melanocortin 5 receptor
Source: EMBO Rep. 2024 Mar 7;25(4):21. doi: 10.1038/s44319-024-00109-6 (PMC11014855; doi:10.1038/s44319-024-00109-6)

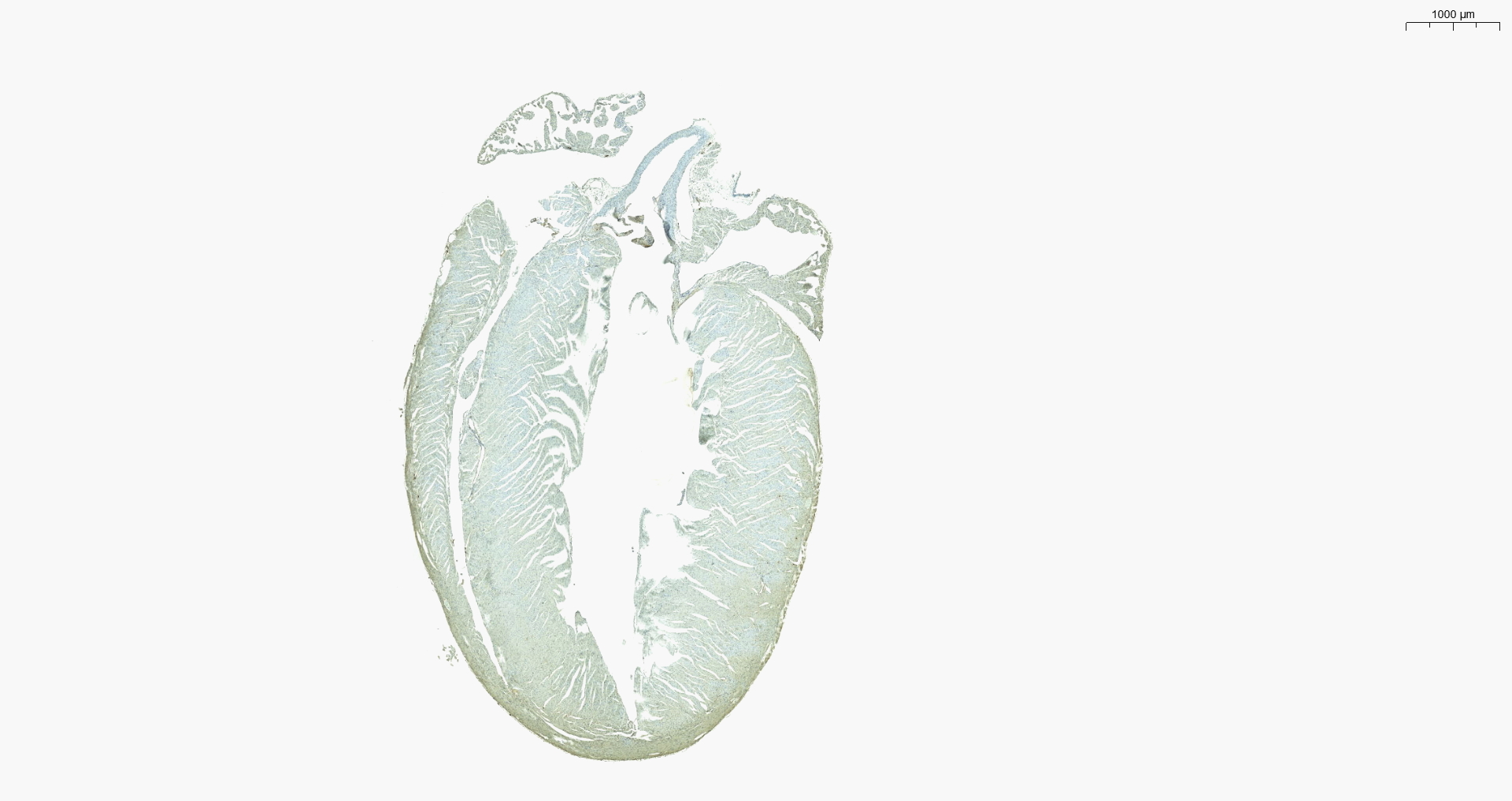

Supplement: Supplementary file 2 — Source Data Fig. 1 [file 44319_2024_109_MOESM2_ESM.zip › Figure 1/Fig 1B microscopic images/Anti-aMSH antibody_1.2x.tiff]

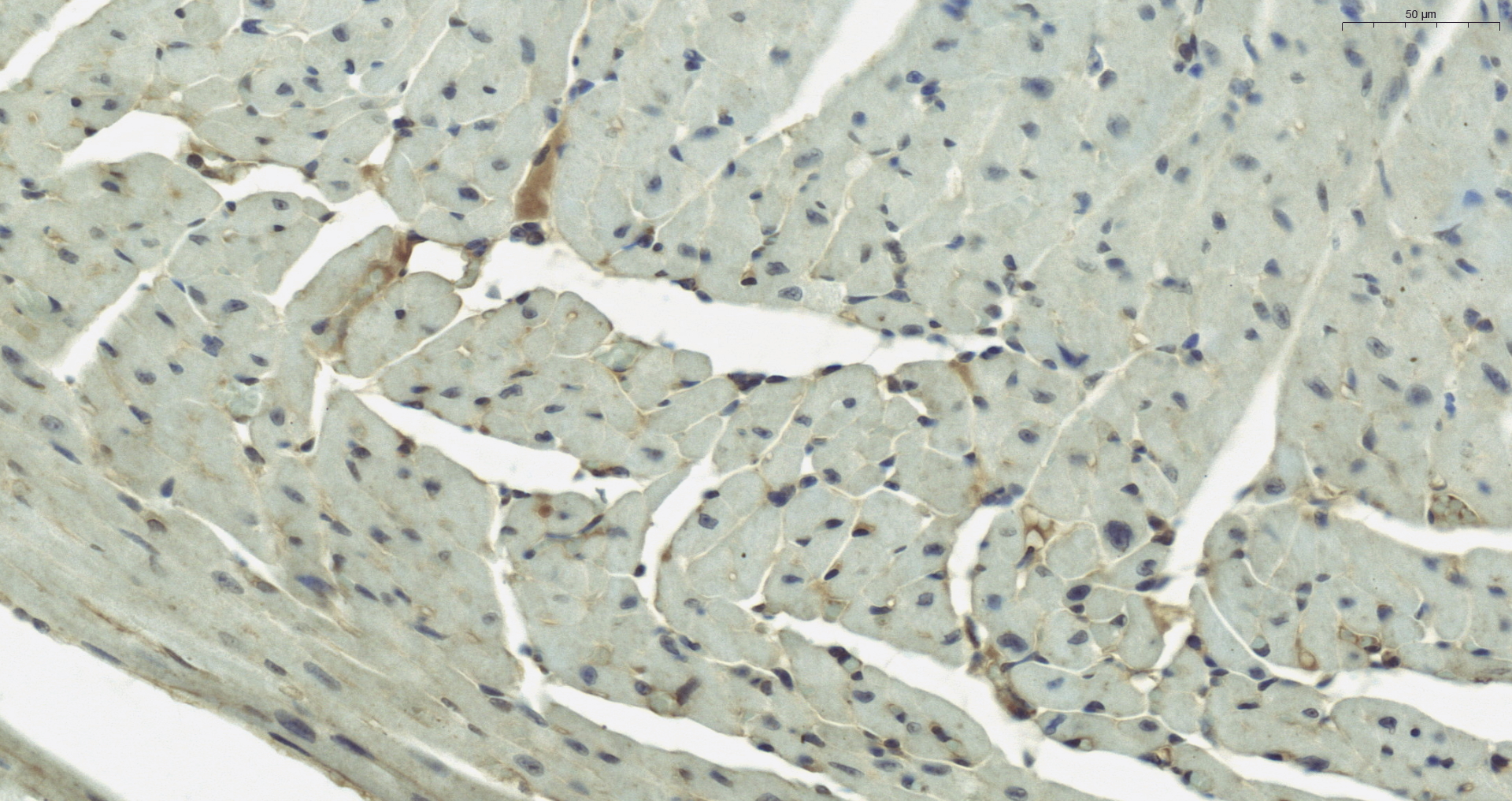

Supplement: Supplementary file 2 — Source Data Fig. 1 [file 44319_2024_109_MOESM2_ESM.zip › Figure 1/Fig 1B microscopic images/Anti-aMSH antibody_40.0x.tiff]

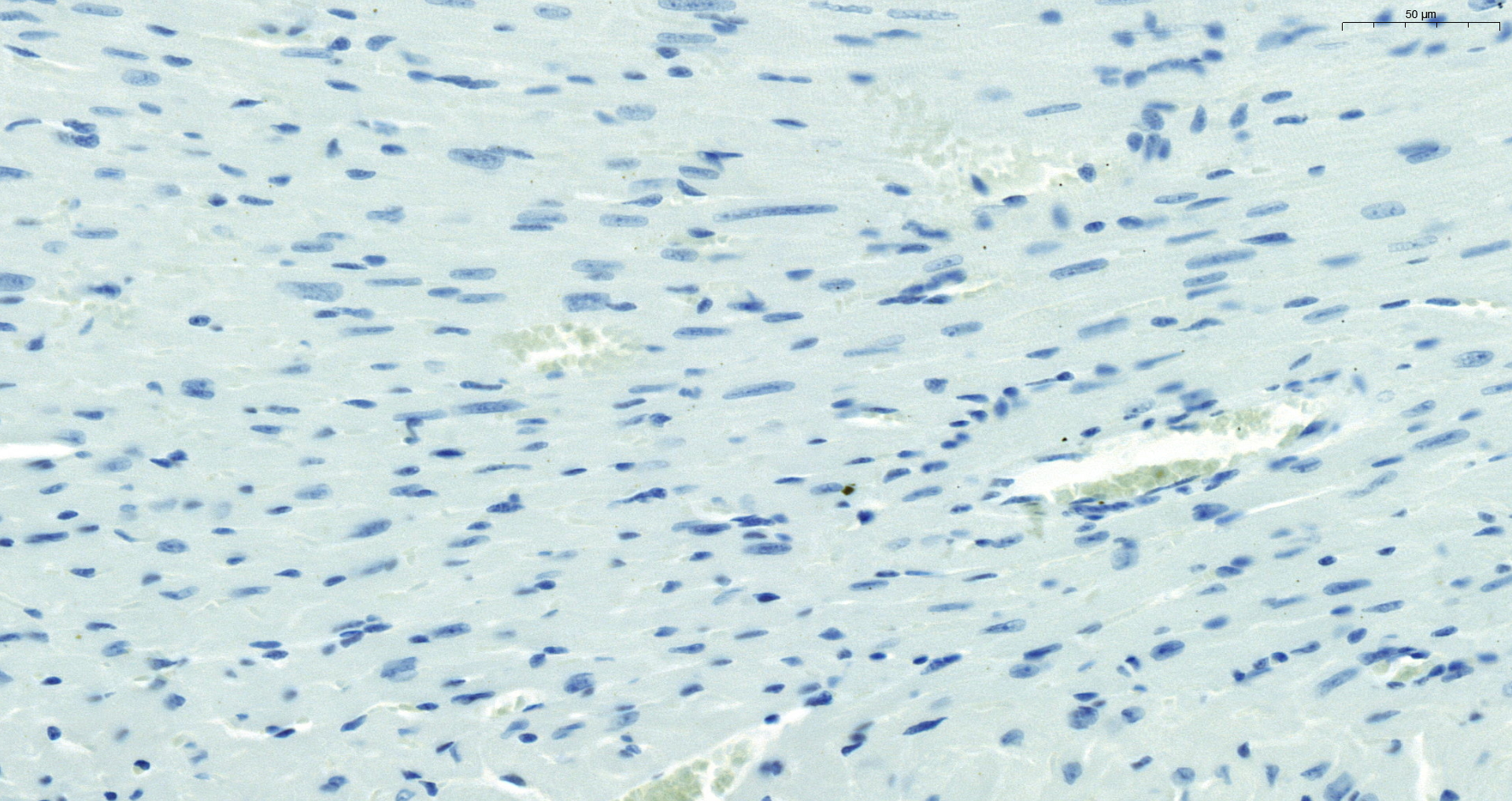

Supplement: Supplementary file 2 — Source Data Fig. 1 [file 44319_2024_109_MOESM2_ESM.zip › Figure 1/Fig 1B microscopic images/Isotype control_40.0x.tiff]

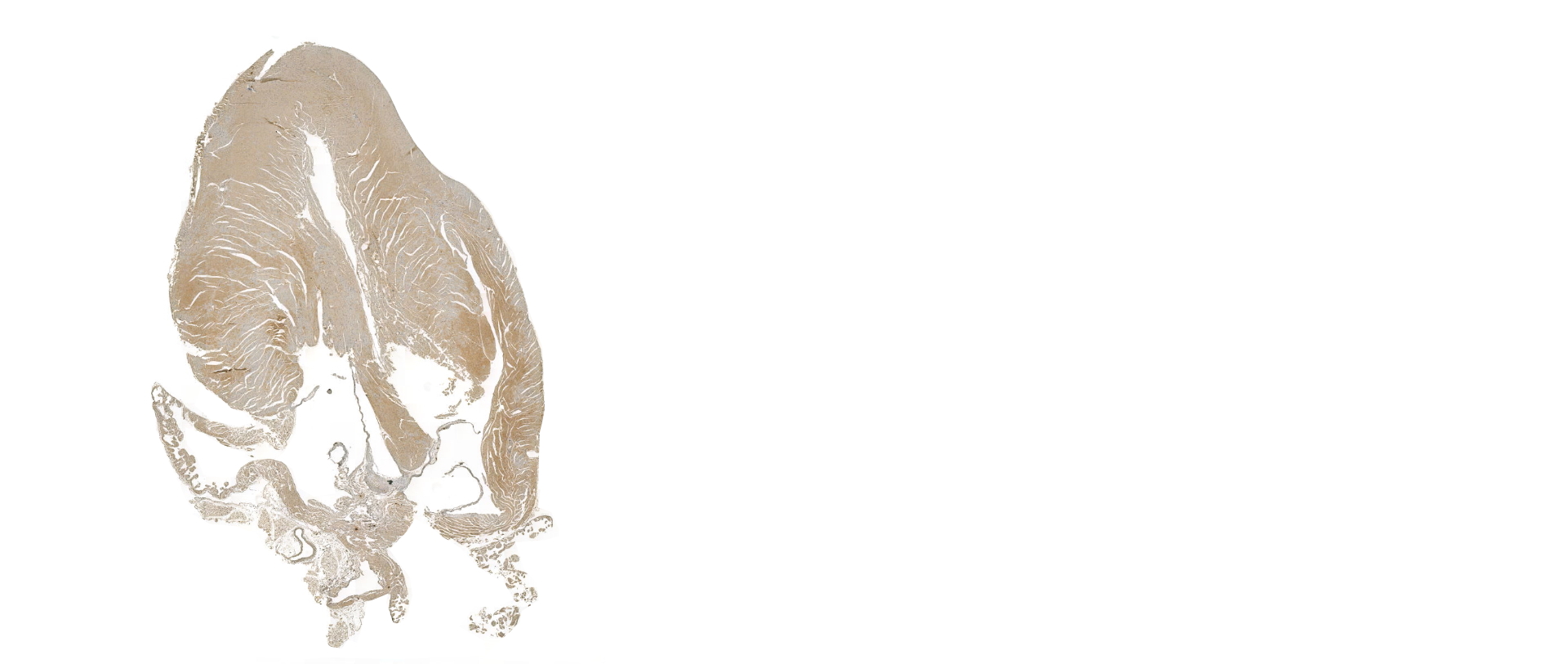

Supplement: Supplementary file 4 — Source Data Fig. 3 [file 44319_2024_109_MOESM4_ESM.zip › Figure 3/Fig 3I microscopic images/Anti-MC5R antibody_0.9x.tiff]

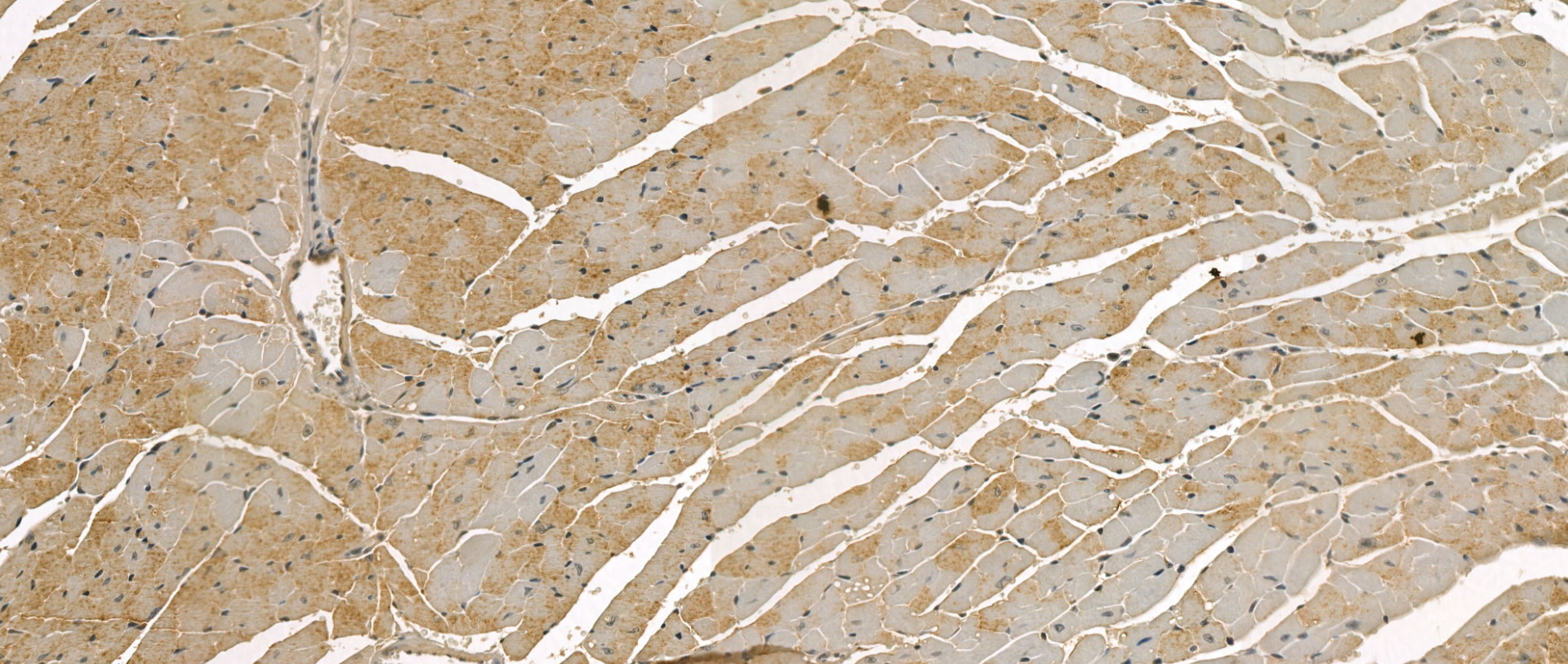

Supplement: Supplementary file 4 — Source Data Fig. 3 [file 44319_2024_109_MOESM4_ESM.zip › Figure 3/Fig 3I microscopic images/Anti-MC5R antibody_20x.tiff]

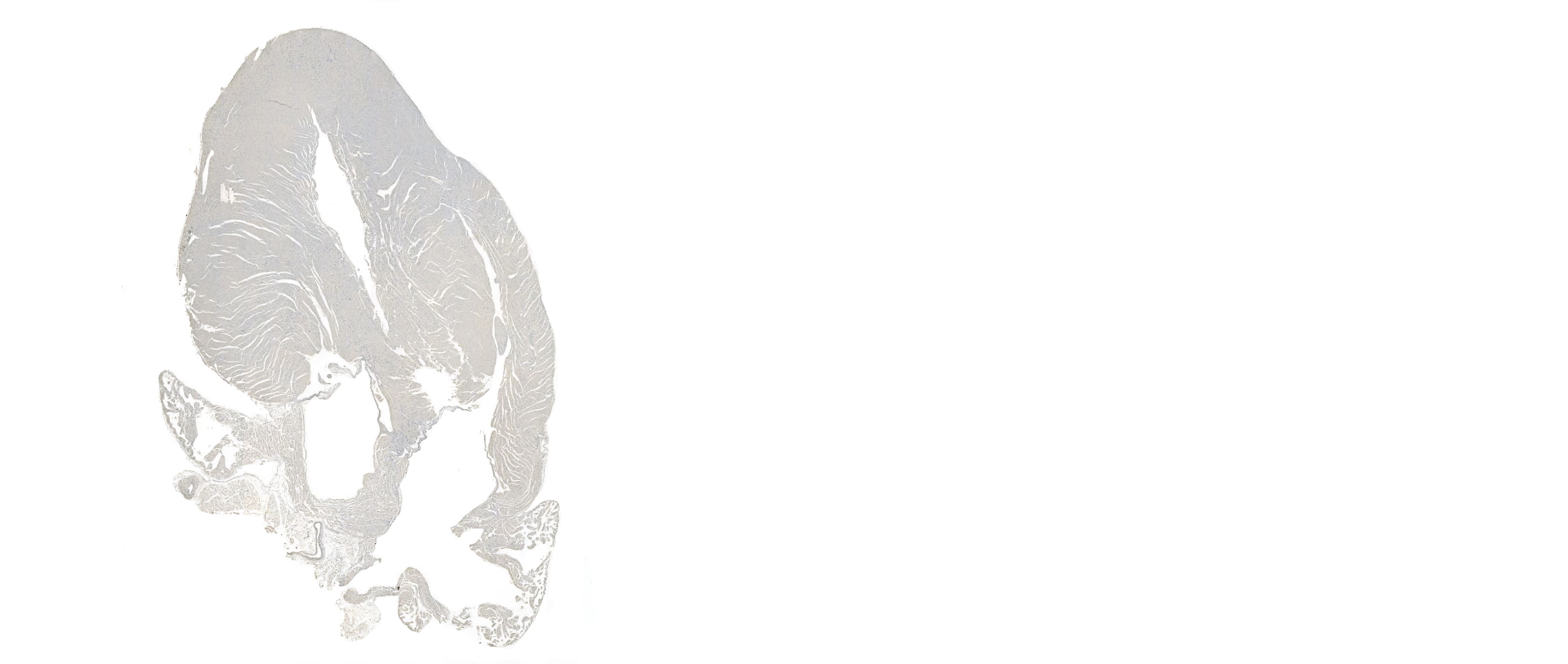

Supplement: Supplementary file 4 — Source Data Fig. 3 [file 44319_2024_109_MOESM4_ESM.zip › Figure 3/Fig 3I microscopic images/Isotype control_0.9x.tiff]

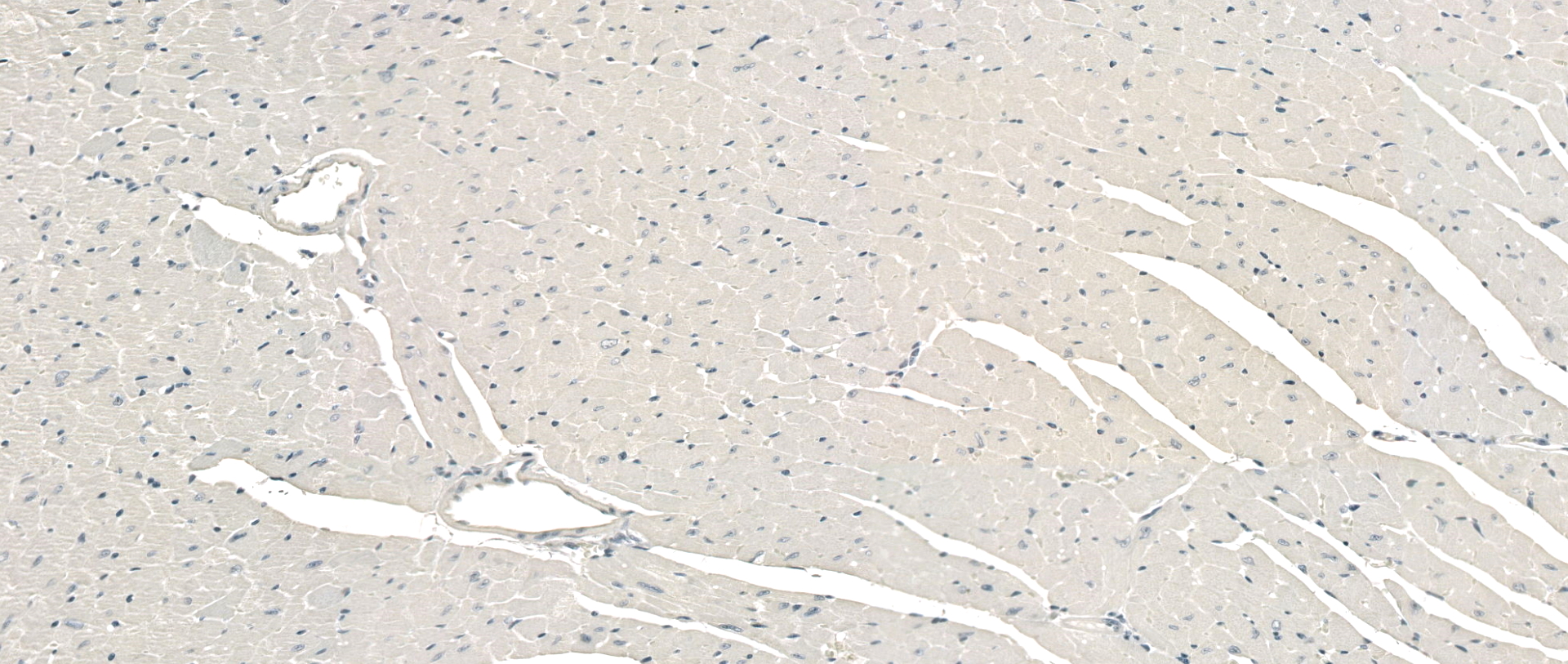

Supplement: Supplementary file 4 — Source Data Fig. 3 [file 44319_2024_109_MOESM4_ESM.zip › Figure 3/Fig 3I microscopic images/Isotype control_20x.tiff]

## Unedited gel for Figure 5H

### Representative lanes marked in red

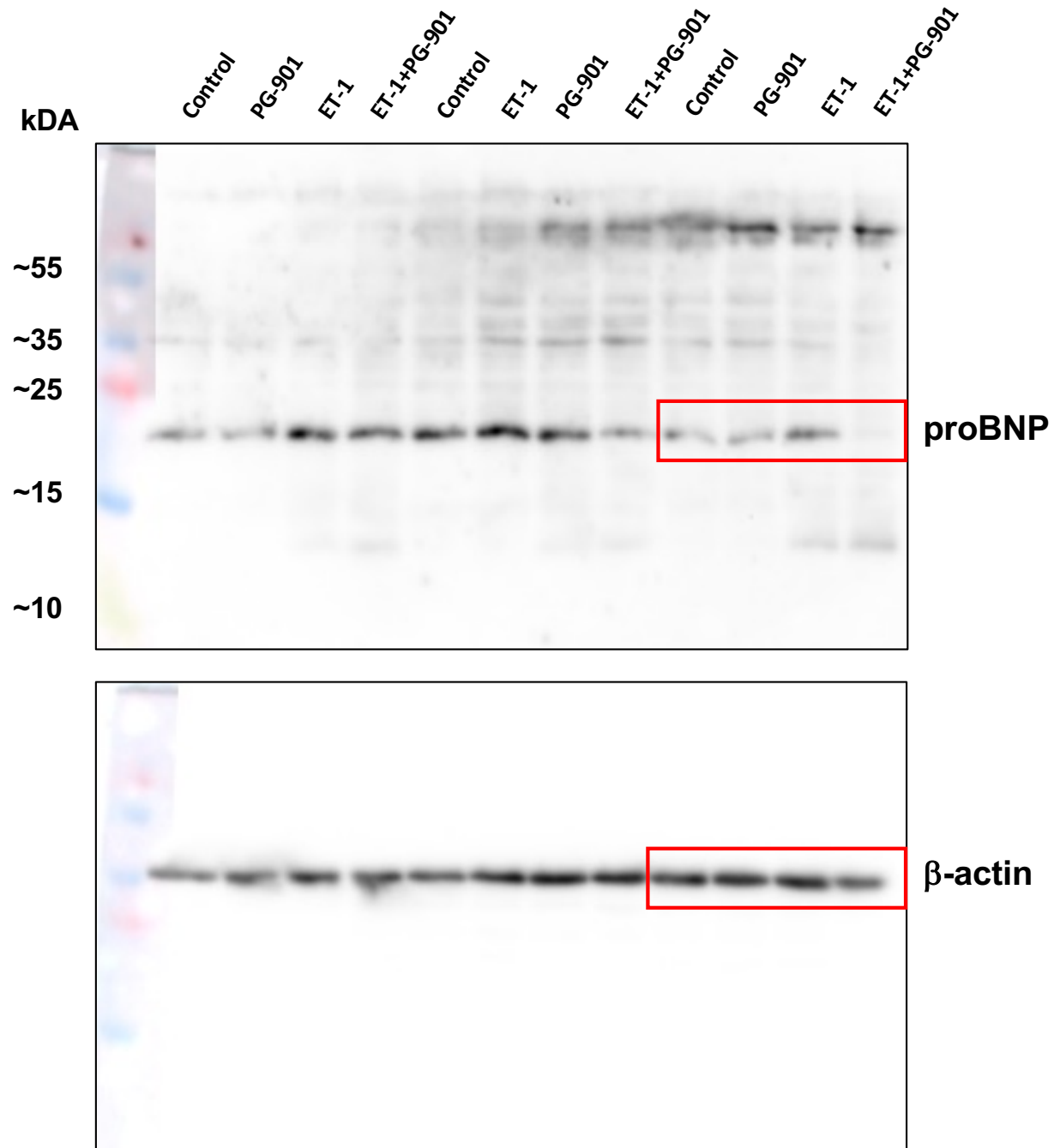

Supplement: Supplementary file 6 — Source Data Fig. 5 [file 44319_2024_109_MOESM6_ESM.zip › Figure 5/Figure 5H_unedited WB image.pdf]
