# Supplementary material for: α-Melanocyte-stimulating hormone alleviates pathological cardiac remodeling via melanocortin 5 receptor
Source: EMBO Rep. 2024 Mar 7;25(4):21. doi: 10.1038/s44319-024-00109-6 (PMC11014855; doi:10.1038/s44319-024-00109-6)

Unedited gel for Figure 3J (left panel)

Representative lanes marked in red

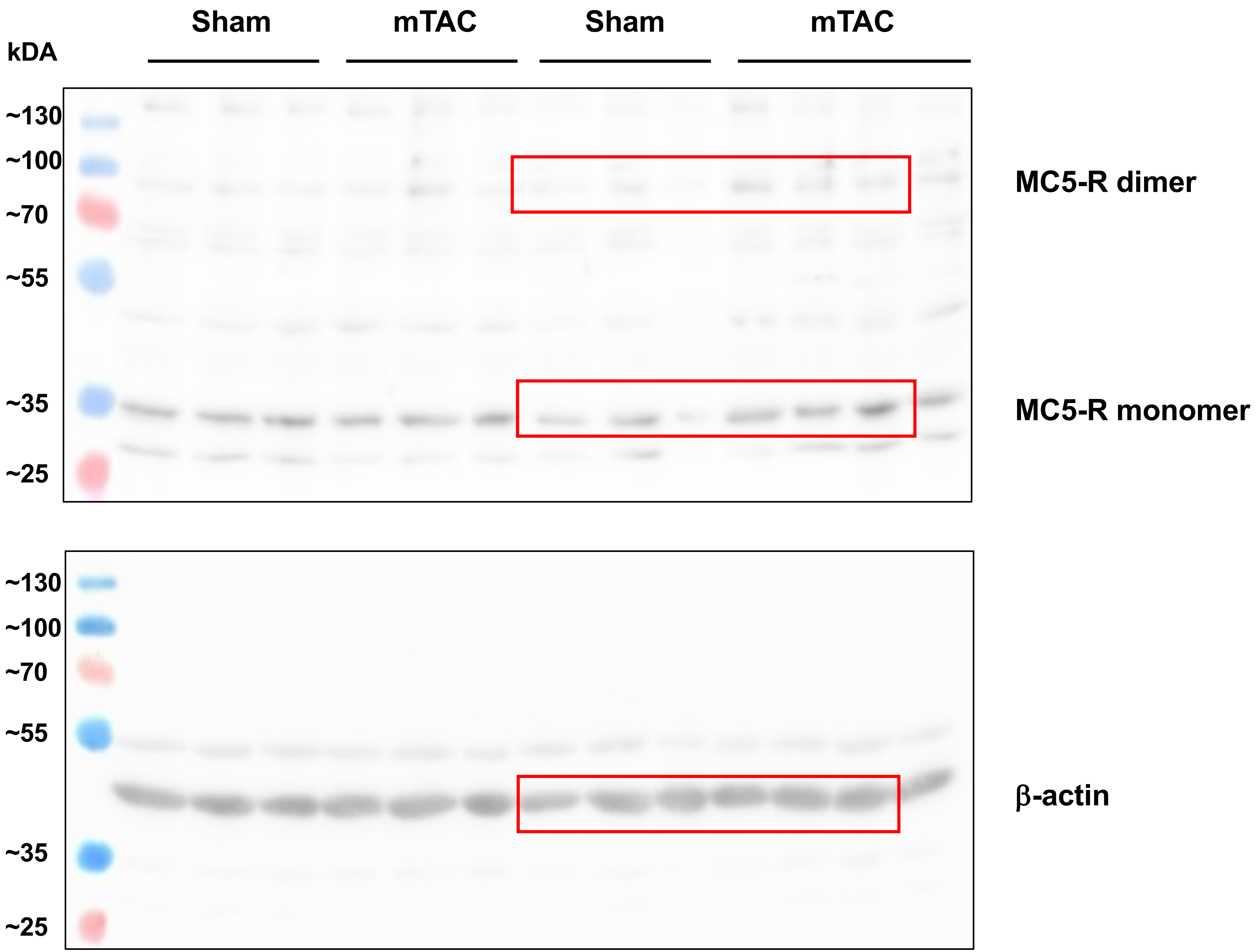

Unedited gel for Figure 3J (right panel)

Representative lanes marked in red

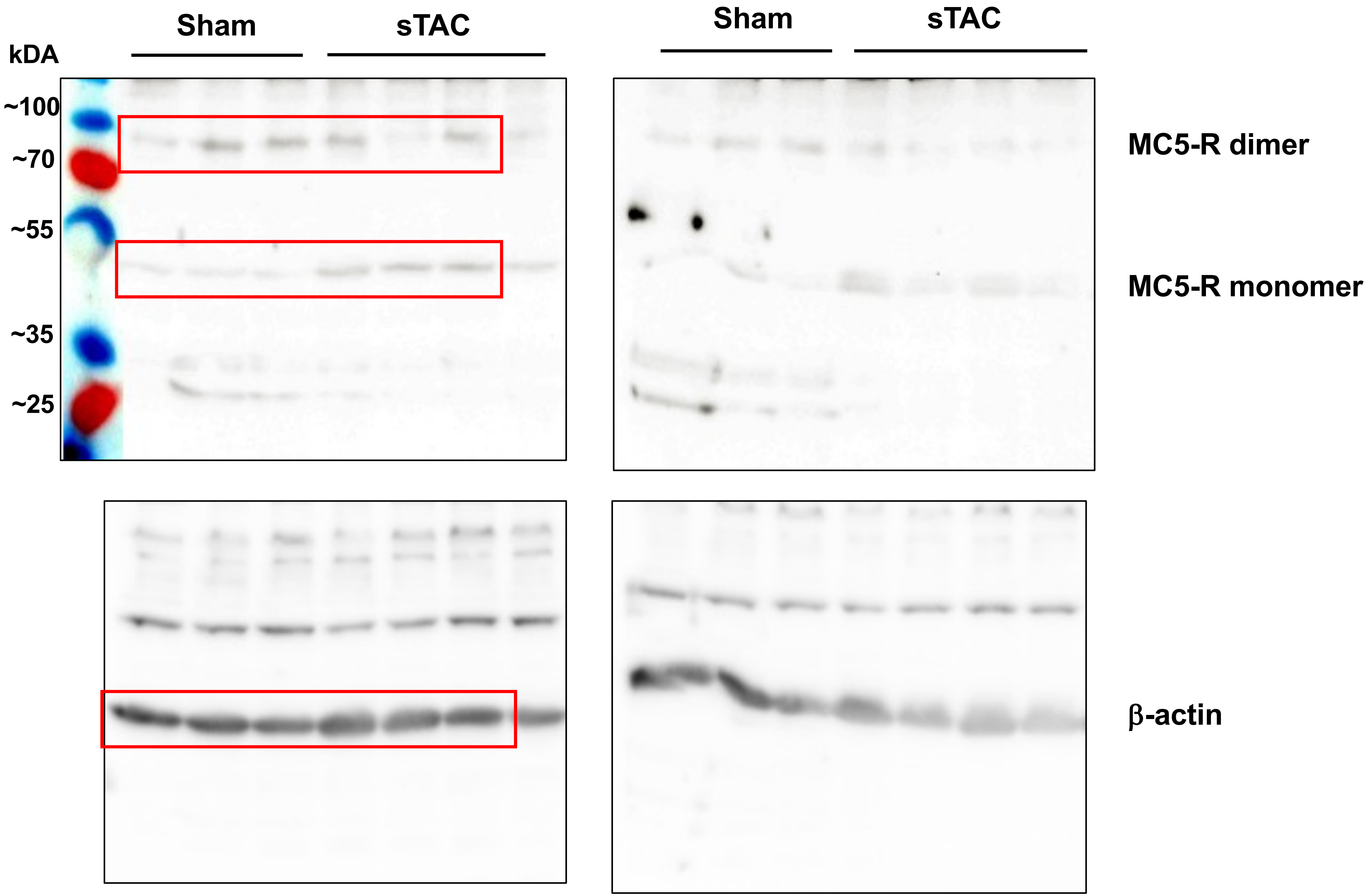

Supplement: Supplementary file 4 — Source Data Fig. 3 [file 44319_2024_109_MOESM4_ESM.zip › Figure 3/Figure 3J_unedited WB images.pdf]
