# Supplementary material for: α-Melanocyte-stimulating hormone alleviates pathological cardiac remodeling via melanocortin 5 receptor
Source: EMBO Rep. 2024 Mar 7;25(4):21. doi: 10.1038/s44319-024-00109-6 (PMC11014855; doi:10.1038/s44319-024-00109-6)
Supplement: Supplementary file 11 — Expanded View Figures [file 44319_2024_109_MOESM11_ESM.pdf]

Expanded View Figures

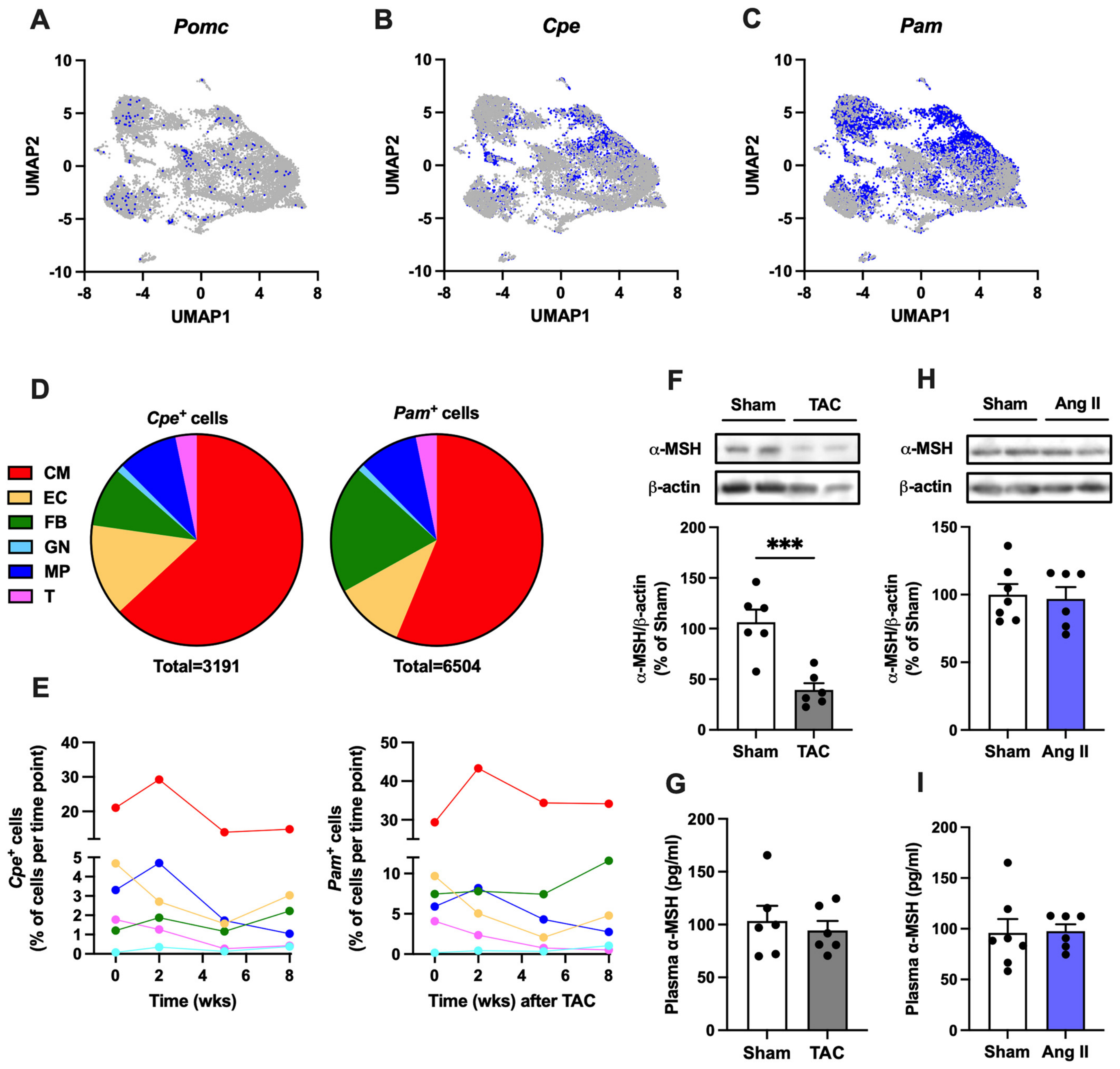

Figure EV1. Single-cell analysis of *Pomc*-, *Cpe*-, and *Pam*-expressing cells in the heart of pressure-overloaded mice.

(**A–C**) Uniform Manifold Approximation and Projection (UMAP) showing 11,492 single cells isolated from C57Bl mice at different stages of cardiac hypertrophy. Blue dots indicate the localization of pro-opiomelanocortin (*Pomc*)-, carboxypeptidase E (Cpe)-α-amidating monooxygenase (*Pam*)-expressing cells in the UMAP-plot. CM indicates cardiomyocyte; EC, endothelial cell; FB, fibroblast; GN, granulocyte; MP, macrophage; and T, T cell. (**D**) Pie charts showing the relative distribution of Cpe^{+}- and Pam^{+} cells in each cell type. (**E**) Changes in the relative amount of Cpe^{+} and Pam^{+} cells in each cell type as a function of time after transverse aortic constriction (TAC) surgery. Pam^{+} and Cpe^{+} cells are expressed as percentage of total number of sequenced at each time point. (**F**) Representative Western blots and quantification of α-MSH (normalized to β-actin) in the LV samples of sham- and TAC-operated mice. $n = 6$ mice per group. ***$P < 0.001$ by Student's t test. (**G**) α-MSH concentration in the plasma of sham- and TAC-operated mice 5 weeks after the surgery. $n = 6$ mice per group. (**H**) Representative Western blots and quantification of α-MSH (normalized to β-actin) in the LV samples of sham-operated and Ang II-infused (4 weeks) mice. $n = 7$ in sham, $n = 6$ in Ang II. (**I**) α-MSH concentration in the plasma of sham-operated and Ang II-infused (4 weeks) mice. $n = 7$ in sham, $n = 6$ in Ang II. Data information: Data are mean ± SEM, each dot represents individual mouse.

 © The Author(s)

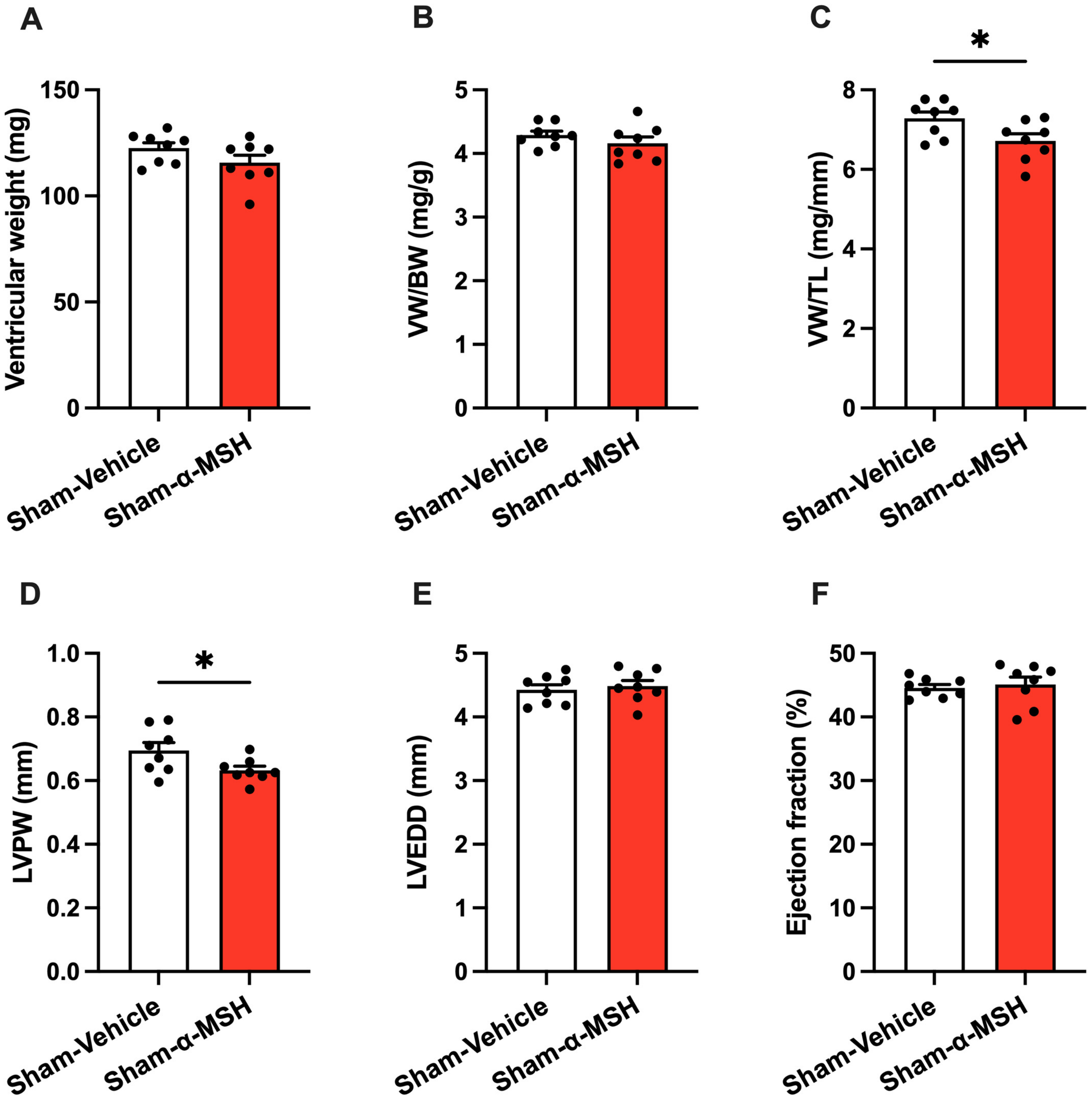

Figure EV2. Ventricular weight and echocardiography in vehicle- and α-MSH-treated mice after 8 weeks of sham operation.

(**A–C**) Ventricular weight, ventricular weight to body weight ratio (VW/BW) and ventricular weight to tibia length ratio (VW/TL) in sham-operated mice treated with either vehicle or α-MSH analog (melanotan II; MT-II). (**D–F**) Left ventricular posterior wall thickness (LVPW), left ventricular end-diastolic dimension (LVEDD), and ejection fraction analyzed by echocardiography at the end of the experiment. Data information: Data are mean ± SEM, $n = 8$ in sham-vehicle and $n = 8$ in sham-α-MSH. *$P < 0.05$ by Student's t test.

© The Author(s)

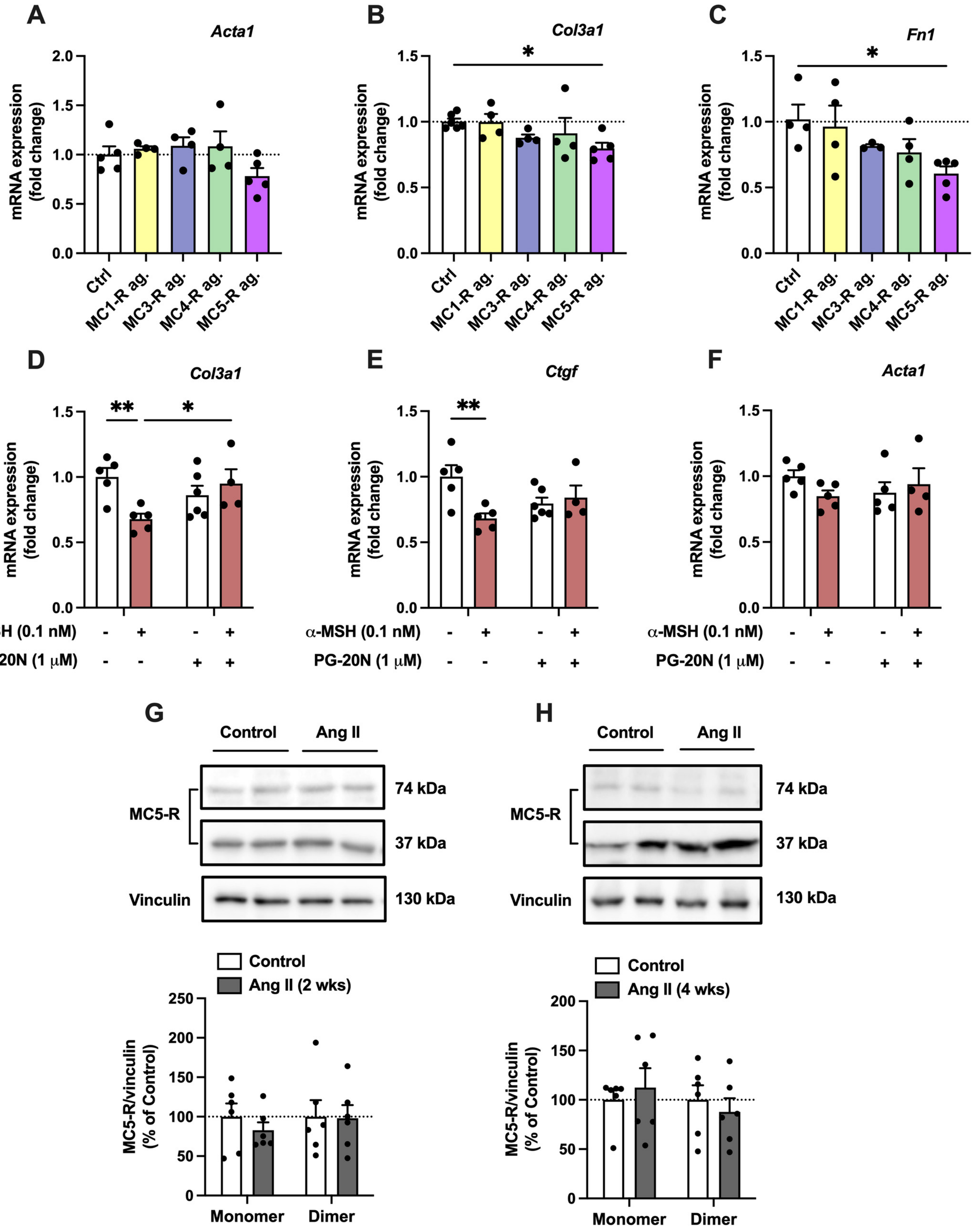
A
Acta1
mRNA expression (fold change)
Ctrl
MC1-R ag.
MC3-R ag.
MC4-R ag.
MC5-R ag.
B
Col3a1
*
C
Fn1
*
D
Col3a1
** *
α-MSH (0.1 nM)
PG-20N (1 μM)
E
Ctgf
**
F
Acta1
G
Control
Ang II
MC5-R
74 kDa
37 kDa
Vinculin
130 kDa
Control
Ang II (2 wks)
MC5-R/vinculin (% of Control)
Monomer
Dimer
H
Control
Ang II
MC5-R
74 kDa
37 kDa
Vinculin
130 kDa
Control
Ang II (4 wks)
MC5-R/vinculin (% of Control)
Monomer
Dimer

 © The Author(s)

Figure EV3. The effects of subtype selective MC-R agonists on gene expression in H9c2 cells and MC5-R expression in hypertrophied mouse heart.

(A–C) *Acta1*, *Col3a1*, and *Fn1* mRNA expression in H9c2 cells treated with MC1-R, MC3-R, MC4-R, or MC5-R selective agonist (10 nM for all agonists) for 3 h. $n = 4–6$ per group (technical replicates) in each graph from 2 independent experiments. (**D–F**) *Nppb*, *Acta2,* and *Tgfb1* expression in H9c2 cells treated with α-MSH (0.1 nM) for 1 h in the absence or presence of the selective MC5-R antagonist PG-20N (1 μM). $n = 4–6$ per group (technical replicates) in each graph from 2 independent experiments. (**G**, **H**) Representative Western blots and quantification of MC5-R monomer and dimer forms (normalized to vinculin) in the LV samples of control, sham-operated mice and Ang II-infused mice. Ang II was infused for 2 (**G**) or 4 weeks (**H**). $n = 5–6$ mice (biological replicates) per group in each graph. Data information: Data are mean ± SEM, $n = 4–6$ per group, *$P < 0.05$ and **$P < 0.01$ for the indicated post hoc comparisons by 1-way ANOVA and Dunnett post hoc tests (**B**, **C**) or by 2-way ANOVA and Bonferroni post hoc tests (**D**, **E**).

© The Author(s)

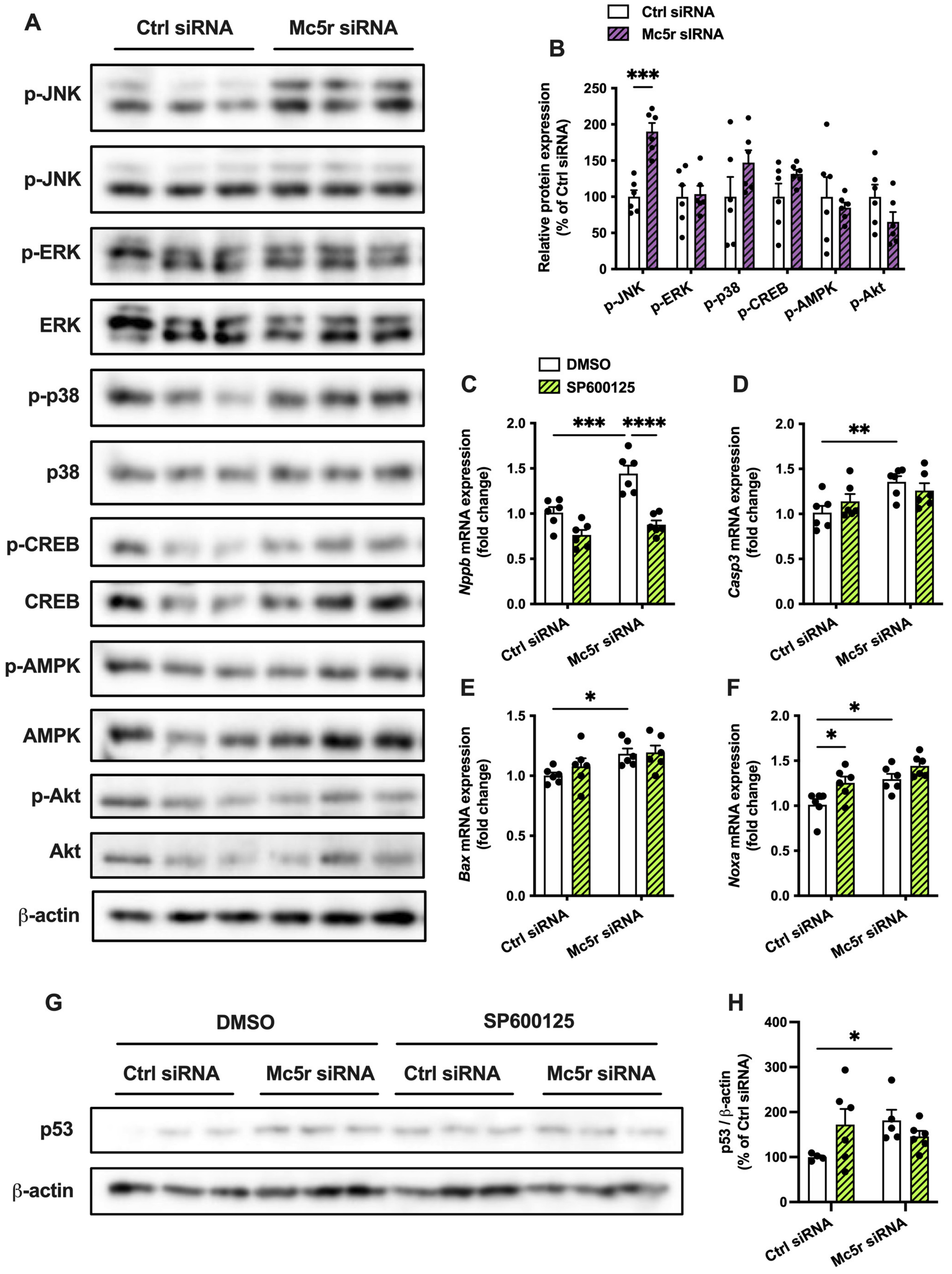
A
Ctrl siRNA
Mc5r siRNA
p-JNK
p-JNK
p-ERK
ERK
p-p38
p38
p-CREB
CREB
p-AMPK
AMPK
p-Akt
Akt
β-actin
B
Ctrl siRNA
Mc5r siRNA
Relative protein expression (% of Ctrl siRNA)
p-JNK
p-ERK
p-p38
p-CREB
p-AMPK
p-Akt
C
DMSO
SP600125
Nppb mRNA expression (fold change)
D
Casp3 mRNA expression (fold change)
E
Bax mRNA expression (fold change)
F
Noxa mRNA expression (fold change)
G
DMSO
SP600125
Ctrl siRNA
Mc5r siRNA
Ctrl siRNA
Mc5r siRNA
p53
β-actin
H
p53 / β-actin (% of Ctrl siRNA)
Ctrl siRNA
Mc5r siRNA

 © The Author(s)

Figure EV4. Mc5r knockdown enhances the expression of pro-apoptotic markers in neonatal mouse ventricular cardiac myocytes (NMCM).

(**A**, **B**) Representative Western blots and quantification of p-JNK (normalized to total JNK), p-ERK (normalized to total ERK), p-p38 (normalized to total p38), p-CREB (normalized to total CREB), p-Akt (normalized to total Akt) and p-AMPK (normalized to total AMPK) in H9c2 cells treated transfected with control siRNA or *Mc5r*-targeting siRNA for 24 h. $n = 6$ (technical replicates) per group from 2 independent experiments. (**C–F**) qPCR analysis of *Nppb*, *Casp3*, *Bax,* and *Noxa* mRNA expression in NMCMs treated with or without the JNK inhibitor SP600125 (10 μM) for 30 min followed by transfection with control siRNA or *Mc5r* targeting siRNA for 24 h. $n = 6$ (technical replicates) per group from 2 independent experiments. (**G**, **H**) Representative Western blots and quantification of p53 (normalized to β-actin) in NMCMs treated with or without the JNK inhibitor SP600125 (10 μM) for 30 min followed by transfection with control siRNA or *Mc5r* targeting siRNA for 24 h. $n = 6$ (technical replicates) per group from 2 independent experiments. Data information: Data are mean ± SEM. $^{*}P < 0.05$, $^{**}P < 0.01$, $^{***}P < 0.001$, and $^{****}P < 0.0001$ for the indicated post hoc comparisons by 2-way ANOVA and Bonferroni post hoc tests (**C–H**).

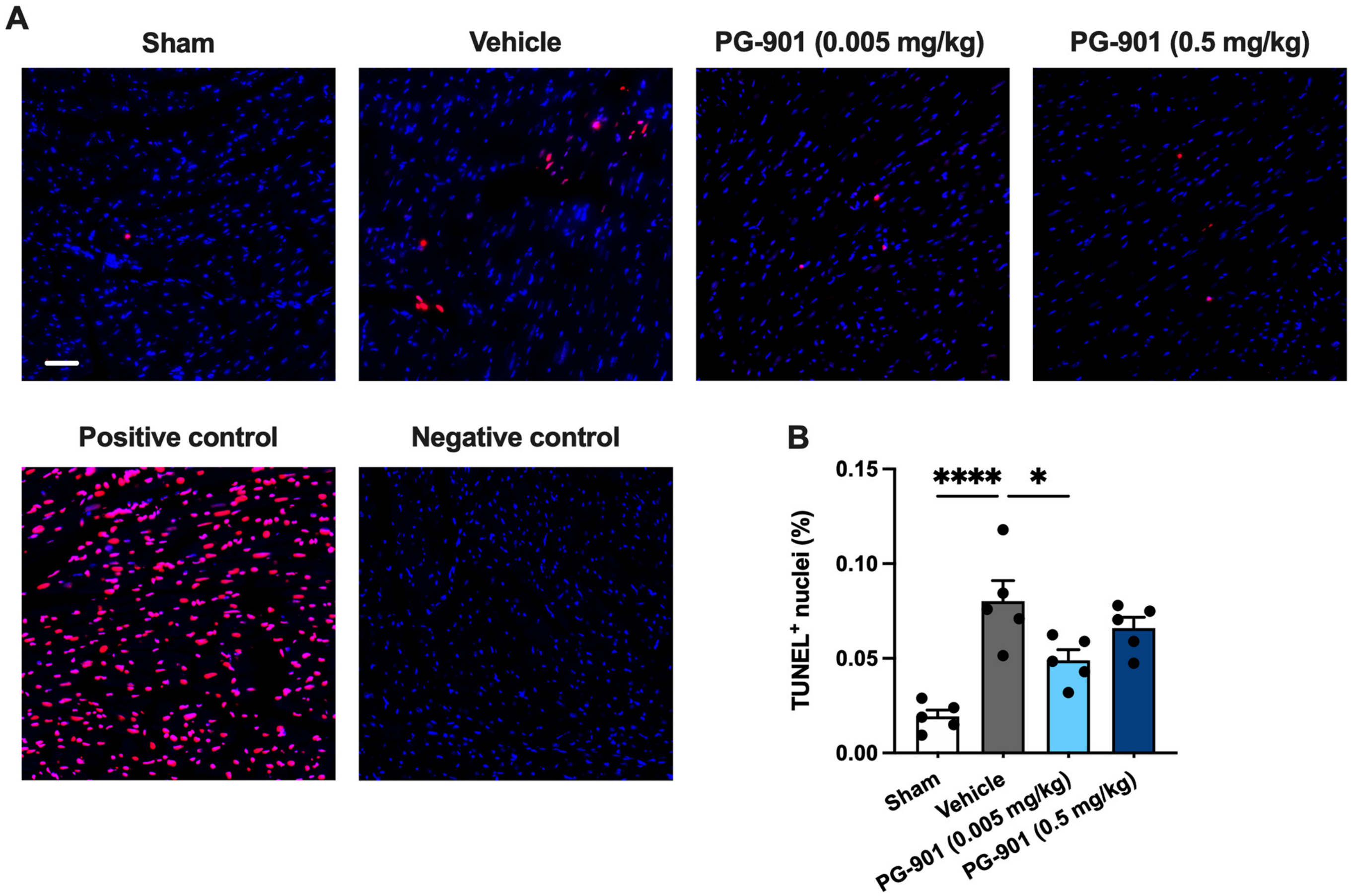

Figure EV5. MC5-R activation with PG-901 reduced the number of apoptotic cells in the heart of TAC-operated mice.

(**A**, **B**) Representative images and quantitative analysis showing relative amount of apoptotic TUNEL-positive nuclei in the LV of sham- and TAC-operated in sham- and TAC-operated mice treated with either vehicle or PG-901 (0.5 or 0.005 mg/kg/day). Scale bar, 50 μm. Positive control was treated with DNase I (0.2 U/μl, 15 min) before being subjected to TUNEL assay. Negative control was treated in a similar way as experimental samples but the labeling was carried out in the absence of TdT enzyme. Data information: Data are mean ± SEM, $n = 5$ mice per group. *$P < 0.05$ and ****$P < 0.0001$ for the indicated comparisons by 1-way ANOVA and Dunnett's post hoc tests.

 © The Author(s)
